# Phytochrome B sets condensate number through graded nucleator states and seeding-site efficacy

**DOI:** 10.1038/s41467-026-73929-w

**Published:** 2026-06-02

**Authors:** Juan Du, Jiangman He, Erik M. Chen, Tingdan Deng, Shivani Talati, Darwin Chang, Theodore J. Mikhail, Meng Chen

**Affiliations:** Department of Botany and Plant Sciences, Center for Plant Cell Biology, Institute for Integrative Genome Biology, University of California, Riverside, CA USA

**Keywords:** Nuclear organization, Light responses, Fluorescence imaging

## Abstract

Photobodies (PBs) are phytochrome B (phyB)-organized nuclear condensates that nucleate at defined subnuclear seeding sites and decrease in number with warming. How cells set PB number—and, more broadly, condensate number—remains poorly understood. Here we show that phyB’s output module (OPM) functions as a universal nucleator for all site-defined PB types, whereas the photosensory module (PSM) attenuates this activity, enabling environmental control. Consistently, missense substitutions in OPM genetically separate intrinsic nucleation potency from PSM-mediated regulation. Unexpectedly, the constitutively active phyB^Y276H^ allele defines a hypoactive nucleation state, supporting graded active conformations with distinct nucleation potency. Temperature tunes PB number through two separable inputs—phyB thermal reversion (nucleator state) and temperature-responsive seeding-site efficacy—whose contributions vary across sites and cell types. Together, these results distinguish nucleation control (number) from growth control (size) and show that condensate number is jointly determined by nucleator state and seeding-site efficacy.

## Abstract (web summary)

Researchers show how plants control the number of nuclear photobodies built by phytochrome B. Photobody number is set by phytochrome B’s activity state and by the strength of nuclear seeding sites, which change with temperature and cell type.

## Introduction

Nuclear bodies are membraneless subnuclear organelles—biomolecular condensates—present in both animal and plant nuclei that compartmentalize and spatiotemporally organize fundamental nuclear functions^1–6^. Studies of canonical nuclear bodies, including nucleoli, Cajal bodies, nuclear speckles, paraspeckles, histone locus bodies, and promyelocytic leukemia protein bodies, have revealed diverse roles in genome organization, transcription, splicing, RNA processing, and DNA replication and repair^1–3^. A prevalent model posits that many membraneless organelles form through phase separation, a concentration-dependent thermodynamic process in which multivalent stereospecific or promiscuous interactions among proteins and/or nucleic acids drive demixing into a dense phase (e.g., nuclear bodies) immersed in a dilute phase (e.g., the nucleoplasm)^4,5^.

Condensate formation typically involves an initiating nucleation step followed by growth^7–9^. In nonliving systems and many in vitro reconstitution settings, nucleation is often described as a homogeneous process in which stochastic assembly of nucleating complexes occurs once bulk concentration exceeds a threshold (critical concentration), after which condensates grow while the surrounding dilute phase is buffered near the critical concentration^7–9^. A main challenge in applying this framework to living cells is explaining whether and how endogenous membraneless organelles nucleate at defined times and locations. Because nucleation sets condensate number, the mechanisms that regulate the number of membraneless organelles in vivo remain poorly understood.

Photobodies (PBs) are light- and temperature-sensing nuclear condensates in plants organized by phytochrome B (phyB), a red (R) and far-red (FR) photoreceptor that also functions as a thermosensor^10–14^. In *Arabidopsis thaliana* (*Arabidopsis*), five phytochromes (phyA-E) are encoded, with phyB serving as a principal detector of R light intensity and spectral quality^15,16^. PhyB is a homodimer composed of an N-terminal photosensory module (PSM) and a C-terminal output module (OPM)^16–18^. The PSM comprises N-terminal extension (NTE), an nPAS (N-terminal Period-Arnt-Single-Minded) domain, a bilin-binding GAF (cGMP-specific phosphodiesterase, Adenylyl cyclase, and FhlA) domain, and the PHY (phytochrome-specific) domain, whereas the OPM contains tandem PAS domains (PAS1/PAS2) and a histidine kinase-related domain (HKRD)^16–18^. PhyB photoconverts between an inactive R-light-absorbing Pr state and an active FR-light-absorbing Pfr state^16–18^. Cryo-electron microscopy (cryo-EM) reveals large Pr-to-Pfr rearrangements that reshape interdomain contacts^19–21^. In Pr, phyB adopts a head-to-tail dimer with extensive PSM-OPM interactions^19^, whereas photoactivation converts phyB into a head-to-head Pfr dimer in which these contacts are relieved^20,21^. Disruption of the PSM-OPM contacts enables downstream outputs in Pfr, including nuclear accumulation, PB formation, and effector interactions^10,19–23^. Importantly, Pfr thermally reverts to Pr at a temperature-dependent rate; warming accelerates thermal reversion and shifts the equilibrium towards Pr, allowing phyB to sense ambient temperature in addition to light^24–27^. Thus, phyB continuously integrates R light intensity, R:FR ratio, and temperature by tuning the Pfr:Pr equilibrium.

PB formation is driven by the active Pfr state^10–12^; accordingly, the steady-state nuclear pattern of phyB-containing PB condensates tracks the Pfr:Pr equilibrium and correlates with phyB-dependent outputs^28–33^. PB dynamics have been characterized primarily using fluorescent protein-tagged phyB (phyB-FP). Under dim R light, phyB-FP remains largely diffuse in the nucleoplasm, whereas above ~0.5 μmol m^−2^ s^−1^ R light it forms discrete condensates^28^. At higher intensities (≥8 μmol m^−2^ s^−1^), nuclei typically contain 2–10 prominent PBs (~0.7–2 μm in diameter)^28,30,31,34^. PhyB is the dominant PB component and provides scaffold-like determinants that recruit selective signaling partners as primary clients (direct phyB binders) and secondary clients (recruited via primaries), including transcription and splicing regulators, components of E3 ubiquitin ligases, kinases/phosphatases, and chaperones^12–14,35–37^. A prominent class of primary clients is the PHYTOCHROME-INTERACTING FACTOR (PIF) family of basic helix-loop-helix transcription factors, including PIF1, PIF3, PIF4, PIF5, PIF7 and PIL1 (PIF2)^12–14,35,36,38–41^. PIFs promote seedling growth programs and act antagonistically to phyB signaling^42–44^. During seedling establishment, PIF1/3/4/5/7 collectively promote hypocotyl elongation by activating growth-associated genes, including those controlling auxin biosynthesis and signaling^42–47^. As light intensity increases, phyB restrains hypocotyl elongation by quantitatively suppressing PIF abundance and activity^12,42–44,48,49^. PBs contribute to this quantitative control by partitioning PIFs between PBs and the surrounding nucleoplasm^12^. Under strong light, a substantial fraction of PIF7 is sequestered to PBs with minimal promoter occupancy; shade dissolves PBs and rapidly restores PIF7 binding and activation of target genes^40^. PBs also sequester PIF5 under strong light; in this case, PB localization additionally stabilizes PIF5 by protecting it from degradation in the surrounding nucleoplasm^39^. Together, these examples support a two-compartment signaling model in which PB dynamics tune nucleoplasmic signaling outputs by modulating the availability of key clients^12^. This raises a central question: how are PB size and number—and thus total client-binding capacity—set and dynamically regulated by light and temperature through changes in phyB photostate?

The overall PB compartmental volume within a nucleus is determined by the number and size of individual PBs. PB formation has been proposed to arise from phase separation of active phyB^32^. While concentration-driven phase separation can account for PB growth, it does not explain how cells set PB number^12,50^. If PB formed solely through bulk phase separation, nucleation would be expected to occur stochastically throughout the nucleoplasm and yield condensates with broadly similar properties. Two key questions therefore are whether PB nucleation is random or site-specific and whether all PB condensates exhibit equivalent biochemical or biophysical states in vivo. Our recent studies indicate that PB nucleation is nonrandom and occurs at defined subnuclear locations^50^. Using chromocenters (CCs) and the nucleolus as landmarks, we found that PBs associate reproducibly with individual CCs, consistent with nucleation at specific chromatin loci^50^. Based on association with the five CCs and the nucleolus, PBs can be classified into 12 site-defined types^50^. Notably, FR light or shade—conditions that shift phyB toward Pr—strongly reduce PB size, whereas moderate warming (from 12 to 27 °C) selectively reduces PB number with little effect on size^31^. Among the nine PB types that occur frequently at 16 °C, six are thermosensitive, with significantly reduced occurrence at 27 °C^50^. Together, these observations indicate that regulated nucleation is a major determinant of PB number and thus PB compartmental volume. Temperature-dependent modulation of PB number therefore provides a powerful entry point to dissect mechanisms controlling condensate nucleation in vivo.

Despite extensive work on PB presence/absence and PB size, the mechanisms that regulate PB number—i.e., PB nucleation at defined sites—remain poorly understood. Prior genetic and cell-based studies indicate that the phyB OPM carries intrinsic determinants for condensation^12^. When expressed separately in *Arabidopsis*, OPM forms nuclear condensates even in darkness, whereas PSM is cytoplasmic; forcing PSM into the nucleus with an ectopic nuclear localization signal (NLS) still yields a diffuse nuclear distribution without condensates^23,32,51^. Similar behavior is observed in HEK293T cells, where only OPM forms condensates^32^. Moreover, an OPM D1040V mutation that disrupts HKRD dimerization abolishes OPM-dependent condensation^52^. Conversely, the constitutively active phyB^Y276H^ allele (YHB) forms PBs irrespective of light^20,53–55^. Together, these results support a model in which OPM provides core condensation activity, whereas PSM restrains OPM in a photostate-dependent manner, rendering PB formation responsive to light and temperature^10,12,23^. However, these studies primarily assess whether condensates form and how large they become; it remains unclear whether OPM-only condensates and YHB PBs correspond to the same site-defined PB types formed by full-length phyB at native nuclear seeding sites.

Here we ask whether OPM can nucleate the full repertoire of site-defined PB types and use OPM—which lacks light/temperature sensing—to separate nucleator-state effects from seeding-site efficacy. We show that OPM alone is sufficient to nucleate all PB types. Comparing steady-state PB occupancy of OPM condensates with full-length phyB PBs reveals that PSM attenuates OPM nucleation in intact phyB. Perturbing PSM conformation by temperature shifts or the constitutively active YHB allele alters PB occupancy, supporting graded active phyB states with distinct nucleation potency; YHB defines a hypoactive state. We further show that temperature tunes PB number through two separable levers—phyB thermal reversion (nucleator state) and temperature-responsive seeding-site efficacy—and that the baseline efficacy and thermosensitivity are cell-type specific, indicating developmental programming of site competence. Together, these results show that PB nucleation is set by integrating environmentally regulated nucleator state with cell-type-programmed seeding-site efficacy.

## Results

### A universal nucleator generates all photobody types

**Fig. 1:**
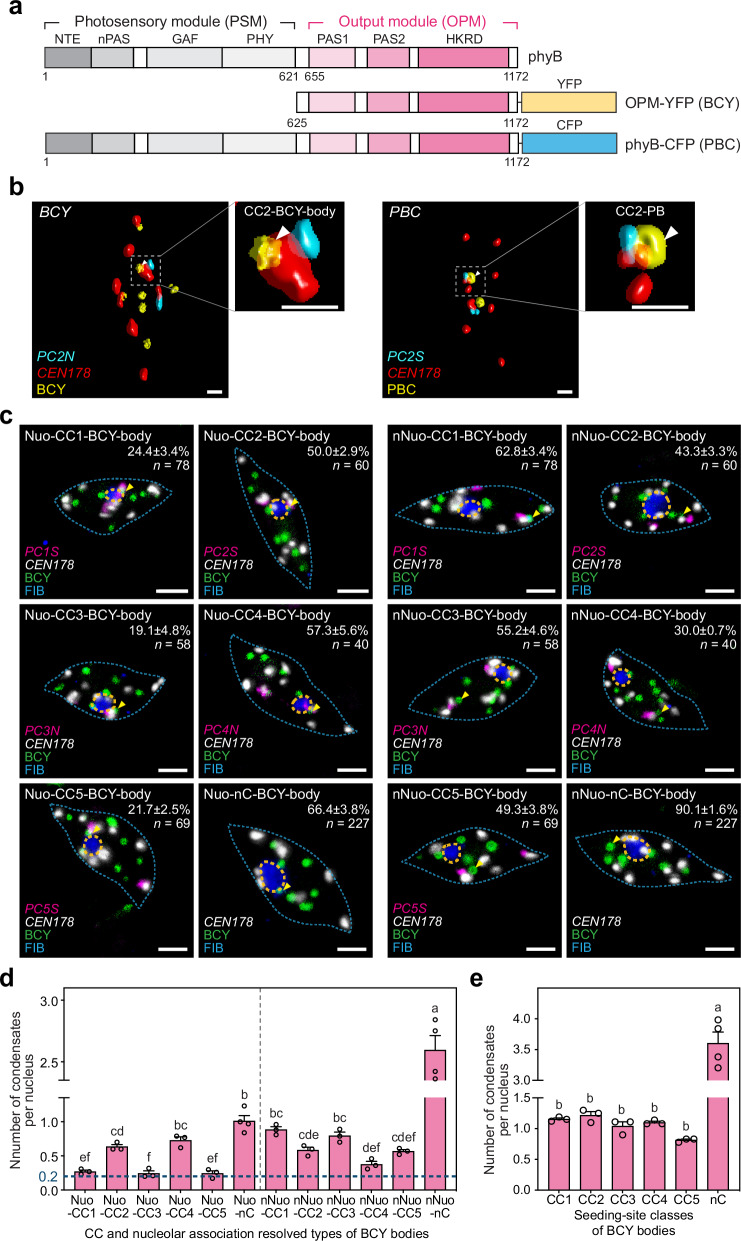
The phyB OPM is sufficient to nucleate all site-defined PB types. **a** Domain architecture of full-length phyB, OPM-YFP (BCY), and phyB-CFP (PBC). The photosensory module (PSM; NTE, nPAS, GAF, PHY) and the output module (OPM; PAS1, PAS2, HKRD) are indicated. **b** Side-by-side representative 3D surface renderings of a CC2-associated condensate formed by BCY (CC2-BCY-body) and by full-length phyB-CFP (PBC photobody; CC2-PB). Insets show the indicated condensate at higher magnification. Chromocenters (CCs; *CEN178*) and the chromosome 2 pericentromeric region (*PC2N* and *PC2S*) are shown in red and cyan, respectively; phyB condensates are shown in yellow. Arrowheads mark the indicated CC2-associated condensate. Scale bars, 1 μm. **c** Representative immunoFISH images showing the twelve site-defined BCY body types in cotyledon pavement-cell nuclei from 4-d-old *BCY* seedlings grown under continuous R light (Rc10; 10 µmol m^−2^ s^−1^) at 16 °C. BCY bodies were detected with anti-GFP (green) and nucleoli with anti-fibrillarin (FIB; blue). CCs (*CEN178*) and chromosome-specific pericentromeric regions (Oligopaints) are shown in white and magenta, respectively. Arrowheads indicate the BCY body type shown. Nuclear and nucleolar boundaries are outlined with dashed blue and orange lines, respectively. For each panel, the percentage of nuclei containing ≥1 indicated BCY body type is shown as mean ± s.e.m. across ≥3 independent biological replicates, and *n* denotes total nuclei scored. Scale bars, 2 μm. **d** Frequency of occurrence (mean number per nucleus) for each of the twelve BCY body types. The blue dashed line at 0.2 per nucleus marks an arbitrary threshold used to visualize frequently (≥0.2) versus rarely (<0.2) observed types. **e** Site-class-level frequency of occurrence of BCY body types grouped by seeding-site classes [CC1-CC5 and non-CC (nC)], pooling nucleolar and non-nucleolar instances. For (**d**, **e**), bars show mean values and error bars indicate ± s.e.m. across independent biological replicates. Differences among groups were assessed by two-sided one-way ANOVA followed by Tukey’s HSD. Exact *p* values are provided in Source Data. The numbers of biological replicates were as follows: CC1-CC5 types (*n* = 3), and nC type (*n* = 4). The source data underlying the quantification of twelve BCY body types in (**c**, **d**) and six seeding-site classes of BCY bodies in (**e**) are provided in the Source Data file.

The phyB OPM forms condensates in plant nuclei and in mammalian cytosol^23,32,51^. The *BCY* line expresses phyB C-terminal OPM fused to YFP (BCY) in the *phyB-9* background (Fig. 1a) and forms condensates (hereafter, BCY bodies) independent of light conditions^23,51^. However, it has remained unclear whether these OPM-only condensates correspond to the site-defined PBs formed by full-length phyB. Full-length phyB-CFP PBs nucleate nonrandomly at twelve defined subnuclear locations in *Arabidopsis* cotyledon pavement-cell nuclei, mapped relative to CCs and the nucleolus^50^. We therefore asked whether OPM alone can nucleate the full repertoire of site-defined PB types at their native nuclear sites.

We performed immunoFISH to co-label BCY bodies (anti-GFP), the nucleolus (anti-fibrillarin), centromeric repeats (*CEN178*), and chromosome-specific pericentromeric regions (Oligopaints)^50^. Three-dimensional rendering showed that CC-associated BCY bodies closely resemble the corresponding CC-associated PBs formed by full-length phyB (Fig. 1b). For quantitative classification, we scored associations using projected images, consistent with established practice for spatial genome/subnuclear organization analyses^56^. BCY bodies segregated into the same twelve site-defined classes previously defined for full-length phyB: five nucleolar CC-associated (Nuo-CC1-5), five non-nucleolar CC-associated (nNuo-CC1-5), nucleolar non-CC (Nuo-nC), and non-nucleolar non-CC (nNuo-nC) (Fig. 1c)^50^. Thus, OPM alone is sufficient to generate all PB types at their characteristic subnuclear sites.

We quantified each type by (i) the fraction of nuclei containing ≥1 body of that type (Fig. 1c) and (ii) the mean number per nucleus (frequency of occurrence) (Fig. 1d). Occurrence varied widely across types. We marked 0.2 per nucleus as an arbitrary threshold—which corresponds to presence in 20% of nuclei and is slightly above our estimated random CC association^50^—distinguishing frequently (≥0.2) from rarely (<0.2) observed types. Notably, nNuo-nC BCY-bodies averaged ~2.5 per nucleus, implying the presence of multiple distinct non-CC, non-nucleolar seeding sites; thus, the nC category likely comprises multiple separable sites, yielding ≥14 site-specific BCY body types when CC/nucleolar stratification is considered.

Because Nuo versus nNuo occupancy at a given CC reflects the fraction of that seeding site positioned adjacent to the nucleolus^50^, we estimated site-class-level occurrence by pooling Nuo and nNuo instances for each CC (CC1-CC5) and for non-CC (nC) BCY bodies (Fig. 1e). The five CC-associated classes showed similar occurrence frequencies, whereas the nC class was higher—consistent with multiple distinct seeding sites grouped within the nC category. Together, these results establish that OPM nucleates condensates at all mapped sites and that CC-proximal sites exhibit comparable seeding efficacy for OPM, with additional competent sites represented in the nC class.

### Regulatory gating attenuates nucleator potency

**Fig. 2:**
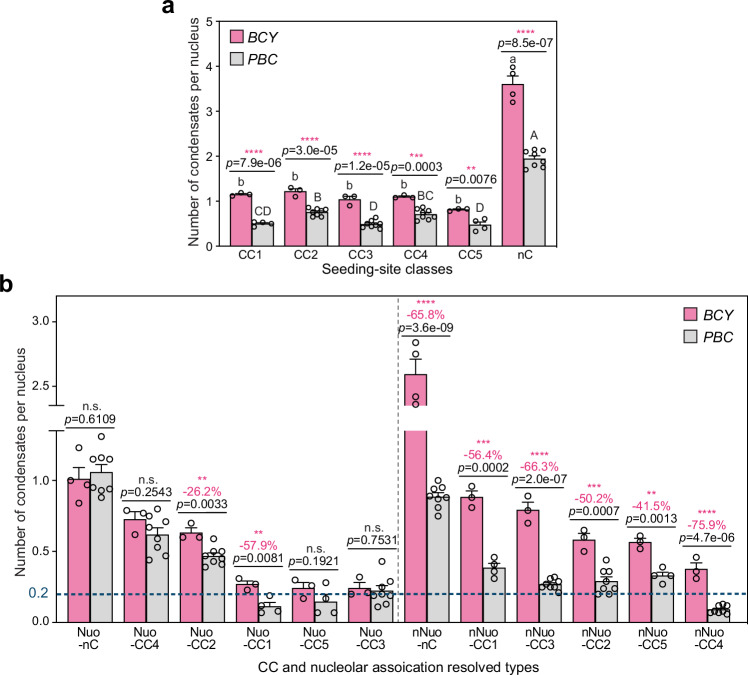
The photosensory module attenuates OPM nucleation in a site-dependent manner. **a** Seeding-site-class frequency of occurrence in *BCY* (OPM-YFP) versus *PBC* (full-length phyB-CFP). Classes are defined by chromocenter association (CC1-CC5) and non-chromocenter (nC); nucleolar and non-nucleolar instances are pooled within each class. Bars show mean values and error bars indicate ± s.e.m. Site-class differences within each genotype were assessed by two-sided one-way ANOVA with Tukey’s HSD. Exact *p* values and test statistics are provided in the Source Data. Asterisks indicate differences between *BCY* and *PBC* within the same class (two-sided Student’s *t*-test; exact *p* values are shown; ** *p* < 0.01, *** *p* < 0.001, **** *p* < 0.0001). **b** Site-resolved occurrence (mean number per nucleus) of the 12 PB types (defined by CC and nucleolar association) in *BCY* versus *PBC*. Bars show mean values and error bars indicate ± s.e.m. Asterisks indicate differences between *BCY* and *PBC* for each condensate type (two-sided Student’s *t*-test; exact *p* values are shown; ** *p* < 0.01, *** *p* < 0.001, **** p < 0.0001; n.s., not significant). The percentage changes between *PBC* and *BCY* are shown for condensate types with significant differences. The blue dashed line at 0.2 per nucleus marks an arbitrary visualization threshold for frequently (≥0.2) versus rarely (<0.2) occurring types. For (**a**, **b**), the numbers of biological replicates (*n*) were as follows: *BCY*, CC1-CC5 types (*n* = 3), and nC type (*n* = 4); *PBC*, CC1 and CC5 types (*n* = 4), and CC2, CC3, CC4, and nC types (*n* = 8). The source data underlying the quantifications of PBC photobodies and BCY bodies in (**a**, **b**) are provided in the Source Data file.

We compared *BCY* (OPM alone) and *PBC* (full-length phyB) at the level of seeding-site classes (CC1-CC5 and nC; nucleolar and non-nucleolar instances pooled). Throughout, we quantify steady-state site occupancy (mean number per nucleus) rather than kinetic nucleation rates, because our imaging captures PB distributions at equilibrium under continuous environmental conditions. All six classes showed lower steady-state occupancy in *PBC* than in *BCY* (Fig. 2a), indicating that PSM attenuates OPM-driven nucleation across all mapped sites. In contrast to *BCY*, *PBC* displayed site-to-site differences, with CC2 and CC4 exceeding other CC classes (Fig. 2a)^50^, consistent with site-dependent regulatory gating in the context of PSM. We next resolved the 12 site-defined PB types (CC identity × nucleolar association). Eight types—including all non-nucleolar classes plus Nuo-CC1 and Nuo-CC2—were reduced in *PBC* relative to *BCY* (Fig. 2b), whereas Nuo-CC3, Nuo-CC4, Nuo-CC5, and Nuo-nC were similar between genotypes (Fig. 2b). Thus, PSM-mediated attenuation is strongest at non-nucleolar sites. Because CC2 and CC4 are preferentially nucleolus-proximal, whereas CC1, CC3, and CC5 are enriched at the nuclear periphery^57^, spatial positioning relative to the nucleolus likely contributes to site-specific attenuation (Fig. 2a, b). As a result, Nuo-CC1, Nuo-CC5, and nNuo-CC4 types shifted from frequently occurring in *BCY* to rarely occurring in *PBC* (Fig. 2b).

### Graded nucleator states with variable nucleation strength

**Fig. 3:**
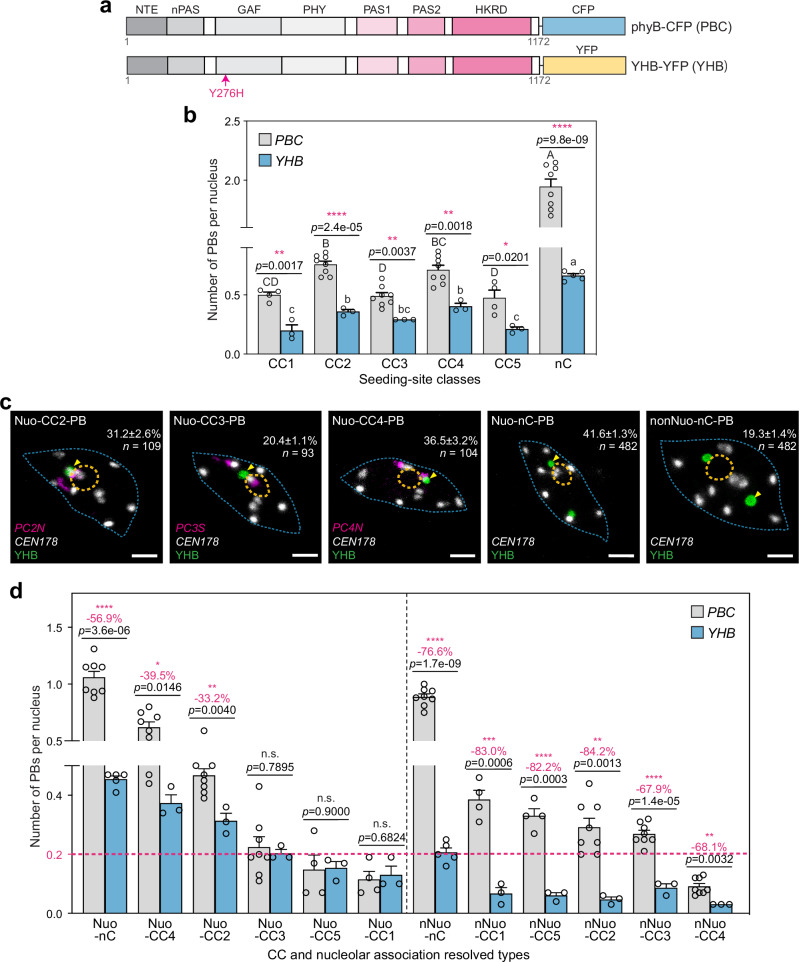
Graded nucleator states encode nucleation strength. **a** Domain maps of full-length phyB-CFP (PBC) and the constitutively active phyB^Y276H^-YFP (YHB). **b** Seeding-site-class occupancy (mean number per nucleus) for *PBC* versus *YHB*, grouped by CC association (CC1-CC5) and non-CC (nC); bars show mean values and error bars indicate ± s.e.m. Within-genotype site-class differences were assessed by two-sided one-way ANOVA with Tukey’s HSD. Exact *p* values and test statistics are provided in Source Data. Asterisks indicate *PBC*-*YHB* differences within the same class (two-sided Student’s *t*-test; * *p* < 0.05, ** *p* < 0.01, *** *p* < 0.001, **** *p* < 0.0001; n.s., not significant; exact *p* values are shown). **c** Representative immunoFISH images showing the five frequently occurring YHB PB types in cotyledon pavement-cell nuclei (4-d-old seedlings, continuous R light 10 µmol m^−2^ s^−1^, 16 °C). YHB PBs were detected with anti-GFP (green); CCs (*CEN178*) and chromosome-specific pericentromeric regions (Oligopaints) are shown in white and magenta, respectively. Arrowheads indicate the PB type shown. Nuclear and nucleolar boundaries are outlined with dashed blue and orange lines, respectively. For each panel, the percentage of nuclei containing ≥1 PB of the indicated type is shown as mean ± s.e.m. across biological replicates; *n* denotes total nuclei scored. Scale bars, 2 μm. **d** Site-resolved occupancy (mean number per nucleus) of the 12 PB types (defined by CC identity and nucleolar association) in *PBC* versus *YHB*. Bars show mean values and error bars indicate ± s.e.m. Asterisks indicate *PBC*-*YHB* differences for each type (two-sided Student’s *t*-test; exact *p* values are shown). The percen*t*age changes between *YHB* and *PBC* are shown for condensate types with significant differences. The dashed magenta line at 0.2 per nucleus marks an arbitrary visualization threshold for frequently (≥0.2) versus rarely (<0.2) observed types. For (**b**, **d**), the numbers of biological replicates (*n*) were as follows: *YHB*, CC1-CC5 types (*n* = 3), and nC type (*n* = 5); *PBC*, CC1 and CC5 types (*n* = 4), and CC2, CC3, CC4, and nC types (*n* = 8). The source data underlying the YHB PB quantifications in (**b**–**d**) are provided in the Source Data file.

To probe how PSM regulates OPM-driven nucleation, we analyzed phyB^Y276H^ allele (YHB), which locks PSM in an active conformation and forms PBs even in darkness (Fig. 3a)^23,53,58,59^. Despite constitutive activity, YHB-YFP forms fewer PBs per nucleus than phyB-CFP^31^, raising the question of whether YHB is broadly impaired or selectively defective at specific seeding sites. We first quantified seeding-site-class occupancy (mean number per nucleus) for CC1-CC5 and nC, pooling nucleolar and non-nucleolar instances. All six classes were reduced in *YHB* relative to *PBC* (Fig. 3b), indicating a global reduction in nucleation potency for YHB across mapped sites.

Resolving the 12 site-defined PB types showed that most were reduced in *YHB* and only five remained frequent (≥0.2 per nucleus): Nuo-CC2, Nuo-CC3, Nuo-CC4, Nuo-nC, and nNuo-nC (Fig. 3c, d). Notably, four of the five are nucleolus-associated, whereas all nNuo-CC types shifted from frequent in *PBC* to rare in *YHB* (Fig. 3d). Thus, YHB defines a hypoactive active state for PB nucleation, with disproportionate attenuation at non-nucleolar sites.

### OPM mutations separate photobody nucleation from regulatory control

Forward genetic screens have identified phyB missense mutations in the OPM that abolish PB formation, but it has been unclear whether these substitutions disrupt PB formation by (i) directly weakening OPM’s intrinsic condensation/nucleation potency or (ii) indirectly perturbing PSM-dependent regulation of OPM. Because PB number is sensitive to both nucleator potency and regulatory gating, OPM mutations provide a tractable avenue to dissect these contributions.

We previously showed that the *phyB-18* allele (HKRD D1040V^60^) disrupts HKRD dimerization and blocks phyB nuclear localization^52^. Consistent with this, OPM-YFP carrying D1040V (BCY^D1040V^) is predominantly cytoplasmic, and addition of an ectopic NLS (BCY^D1040V^-NLS) restores nuclear localization but yields diffuse nucleoplasmic signal without condensates^52^. Thus, D1040V compromises OPM-driven PB condensation in addition to impairing nuclear accumulation.

**Fig. 4:**
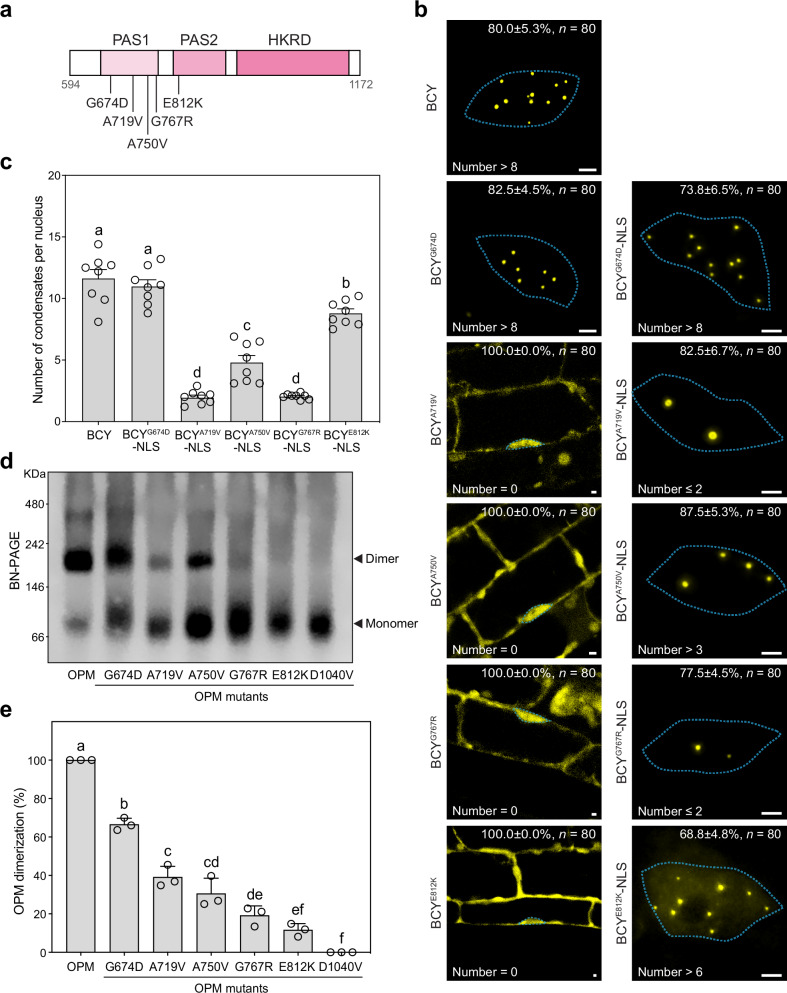
OPM mutations dissociate nucleation potency from regulation. **a** Positions of the indicated amino-acid substitutions (G674D, A719V, A750V, G767R, and E812K) within the phyB output module (OPM). **b** Maximum-projection, deconvolved fluorescence images showing representative steady-state nuclear BCY body patterns in 4-d-old seedlings grown under continuous R light (10 µmol m^−2^ s^−1^) at 16 °C. *BCY* (OPM-YFP) and *BCY*^*G674D*^ were imaged in cotyledon pavement cell nuclei, *BCY*^*A719V*^, *BCY*^*A750V*^, *BCY*^*G767R*^, and *BCY*^*E812K*^ were imaged in hypocotyl epidermal cells, because these mutants showed weak signals in cotyledon epidermal cells under identical imaging settings. The corresponding NLS-tagged variants (*BCY*^*G674D*^*-NLS*, *BCY*^*A719V*^*-NLS*, *BCY*^*A750V*^*-NLS*, *BCY*^*G767R*^*-NLS*, and *BCY*^*E812K*^*-NLS*) were imaged in cotyledon pavement cells. Nuclear boundaries are outlined with dashed blue lines. For *BCY*^*A719V*^, *BCY*^*A750V*^, *BCY*^*G767R*^, and *BCY*^*E812K*^, representative fluorescence images are shown to indicate nuclear and cytoplasmic localization. For each genotype, the percentage of nuclei displaying the indicated pattern is reported as mean ± s.e.m. with total nuclei scored (*n*). Scale bars, 2 μm. **c** Condensates per nucleus for *BCY* and *BCY-NLS* mutants (conditions as in **b**). Bars show mean values and error bars indicate ± s.e.m. (*n* = 8 biological replicates). Differences in condensate number among genotypes were assessed by two-sided one-way ANOVA with Tukey’s HSD; exact *p* values and test statistics are provided in Source Data. **d** Immunoblot analysis of OPM dimerization. In vitro-translated HA-tagged OPM and OPM mutant proteins were resolved by blue-native PAGE (BN-PAGE) and detected with anti-HA. **e** Quantification of OPM dimerization [dimer/(dimer + monomer)], normalized to wild-type OPM (100%). Data are presented as mean ± s.d. (*n* = 3 biological replicates). Differences among variants were assessed by two-sided one-way ANOVA with Tukey’s HSD, exact *p* values and test statistics are provided in Source Data. Source data for condensate quantifications (**b**, **c**) and immunoblots (**d**, **e**) are provided in the Source Data file.

Here we analyzed five additional OPM substitutions (Fig. 4a): G674D, A719V, A750V, G767R, and E812K. G674D, A719V, and E812K were recovered as PB-deficient mutants in a forward screen, where full-length phyB-GFP carrying each mutation is diffuse in the nucleoplasm^28^. A750V and G767R were identified as strong loss-of-function mutations for phyB activity^61^. phyB^G767R^-GFP is predominantly cytoplasmic, consistent with impaired nuclear accumulation^51^. Because OPM contains determinants for both nuclear accumulation and condensation, we tested each substitution in two contexts: BCY (OPM-YFP), which reports the combined outcome of intrinsic nuclear accumulation plus condensation, and BCY-NLS, which bypasses intrinsic OPM nuclear localization and more directly reports nuclear condensation competence.

In the BCY context, the substitutions fell into two groups (Fig. 4b). BCY^A719V^, BCY^A750V^, BCY^G767R^, and BCY^E812K^ showed mixed nuclear/cytoplasmic signals, consistent with impaired nuclear accumulation. In contrast, BCY^G674D^ localized predominantly to nuclear condensates, resembling wild-type BCY, indicating that G674D does not strongly compromise OPM nuclear accumulation or condensate formation in the OPM-only setting.

In the BCY-NLS context, all five variants entered the nucleus and formed BCY bodies, but with distinct condensate numbers per nucleus (Fig. 4b, c), indicating differences in condensation nucleation. BCY^G674D^-NLS produced condensate numbers comparable to those of BCY, whereas A719V, A750V, G767R, and E812K each reduced condensate numbers to varying degrees. Together, these data indicate that A719V, A750V, G767R, and E812K compromise OPM function at two levels—nuclear accumulation (BCY) and BCY-body number, even when nuclear localization is enforced (BCY-NLS)—consistent with reduced nuclear localization/retention and PB nucleation potency.

By contrast, G674D behaved like wild-type OPM in both BCY and BCY-NLS contexts, yet was reported to impair PB formation in full-length phyB^28^. This discrepancy suggests that G674D primarily affects PSM-OPM coupling in full-length phyB, rather than abolishing the intrinsic condensation competence of OPM.

To test whether condensation phenotypes correlate with OPM dimerization, we analyzed in vitro-translated OPM variants by blue-native PAGE (BN-PAGE), using OPM^D1040V^ as a low-dimerization control (Fig. 4d, e)^52^. All five substitutions reduced dimerization relative to wild-type OPM, but each retained higher dimerization than D1040V. G674D showed the highest residual dimerization (>60% of wild-type), consistent with its preserved condensation competence (Fig. 4b–e). However, dimerization did not strictly correlate with condensate number: E812K exhibited the lowest dimerization among the five substitutions (<10% of wild-type) yet retained higher condensate output than A719V, A750V, or G767R. Thus, OPM-driven condensation nucleation in vivo is not determined solely by OPM dimerization and likely depends on additional OPM activities and/or interactions.

Together, these results identify OPM residues that preferentially impact intrinsic condensation nucleation potency versus those that likely perturb regulatory coupling in full-length phyB, providing genetic separation between PB nucleation and its regulation.

### Temperature tunes photobody number via nucleator state and site efficacy

**Fig. 5:**
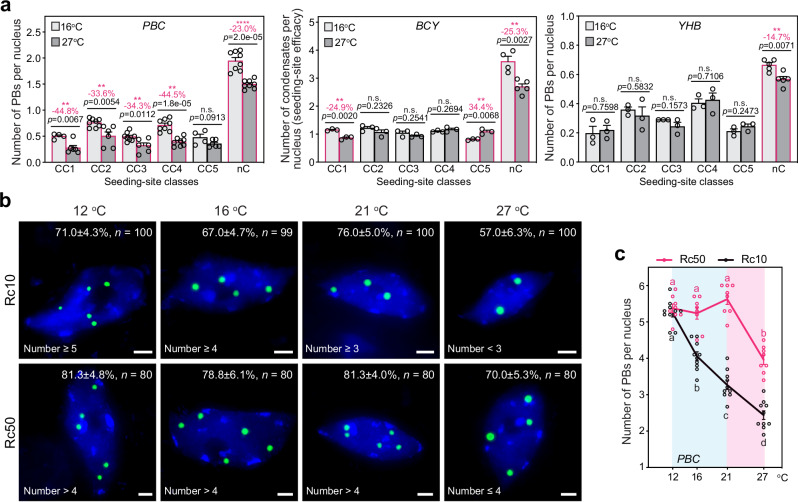
Temperature controls condensate number through two separable levers. **a** Seeding-site class occupancy (mean number per nucleus) for PBC (phyB-CFP) PBs, BCY (OPM-YFP) bodies, and YHB PBs at 16 and 27 °C in 4-d-old seedlings grown under continuous red light 10 µmol m^−2^ s^−1^ (Rc10). Classes are defined by chromocenter association (CC1-CC5) and non-chromocenter (nC); nucleolar and non-nucleolar instances are pooled within each class. Because OPM lacks light/temperature sensing, temperature-dependent changes in BCY occupancy report temperature-responsive seeding-site efficacy. Bars show mean values and error bars indicate ± s.e.m. The numbers of biological replicates (*n*) were as follows: *PBC* 16 °C, CC1/CC5 types (*n* = 4) and CC2-CC4/nC (*n* = 8); *PBC* 27 °C, CC1-CC3/CC5 (*n* = 6) and CC4/nC (*n* = 8); *BCY* 16 °C, CC1-CC5 (*n* = 3) and nC (*n* = 4); *BCY* 27 °C, CC1-CC5 (*n* = 3) and nC (*n* = 5); *YHB* 16 and 27 °C, CC1-CC5 (*n* = 3) and nC (*n* = 5). Within-genotype temperature effects (16 vs 27 °C) are indicated by asterisks (two-sided Student’s *t*-test; ** *p* < 0.01, **** *p* < 0.0001; n.s., not significant); exact *p* values are shown. Percentage changes between 27 °C and 16 °C are shown for those with significant differences. **b** Separating phyB-thermal-reversion-dependent and -independent effects. Maximum-projection fluorescence images showing representative steady-state phyB-CFP PBs in cotyledon epidermal pavement-cell nuclei of 4-d-old *PBC* seedlings grown at 12, 16, 21, or 27 °C under Rc10 or saturating R light (Rc50; 50 µmol m^−2^ s^−1^). Nuclei are counterstained with DAPI (blue). For each condition, the percentage of nuclei displaying the representative PB pattern is shown as mean ± s.e.m. across 8 biological replicates under Rc10 and Rc50; *n* denotes total nuclei scored. Scale bars, 2 μm. **c** Global PB occupancy (mean PBs per nucleus) in *PBC* under the conditions in (**b**). Bars show mean values and error bars indicate ± s.e.m. across 10 biological replicates under Rc10 and 8 biological replicates under Rc50. Differences among temperatures within each light condition were assessed by two-sided one-way ANOVA with Tukey’s HSD; exact *p* values and test statistics are provided in Source Data. Under Rc10, PB occupancy decreases from 12 to 21 °C and further from 21 to 27 °C. Under Rc50, the 12-to-21 °C decrease is abolished (consistent with saturation of thermal-reversion effects), whereas the 21-to-27 °C decrease persists, indicating a thermal-reversion-independent component consistent with regulation of seeding-site efficacy. Source data for **a** (site-class occupancy at 16 and 27 °C) and **b**, **c** (PB number under Rc10 and Rc50) are provided in the Source Data file.

PB number depends on phyB conformation, which is controlled by light-driven photoconversion and temperature-dependent thermal reversion. We previously showed that temperature increases from 12 to 27 °C progressively reduce PB number by eliminating a subset of PBs, indicating that PB occupancy is thermosensitive^31,50^. We asked whether this thermosensitivity reflects only phyB thermal reversion (a nucleator-state effect) or also temperature-dependent changes in seeding-site efficacy. Comparing PBC and BCY across CC-defined classes (CC1-CC5 and nC) revealed three distinct patterns (Fig. 5a). First, in PBC, occupancy decreased with warming for all classes except CC5, showing broad thermosensitivity in full-length phyB. Second, for CC2, CC3, and CC4, BCY occupancy was temperature-insensitive, indicating that the corresponding PBC decreases are driven primarily by phyB thermal reversion. Third, CC1 and nC showed reduced BCY occupancy at 27 °C, revealing sites whose seeding efficacy itself declines with warming, implying combined nucleator-state and site-efficacy contributions to PBC. Conversely, CC5 showed stable occupancy in PBC but increased BCY occupancy with warming, suggesting opposing inputs: thermal reversion reduces nucleator potency while CC5 seeding efficacy increases. Consistent with this site-efficacy interpretation, among frequently occurring YHB classes (CC2, CC4, nC), only nC decreased with warming, again pointing to a site-dependent efficacy change in the absence of thermal reversion in YHB.

Temperature responses under continuous light integrate phyB-dependent and phyB-independent thermosensing pathways^26^. Prior work shows that the 12-to-21 °C response depends primarily on phyB thermal reversion, whereas the 21-to-27 °C response integrates additional pathways, including chloroplast-sucrose-mediated and ELF3-dependent mechanisms^26^. Moreover, when R light reaches saturating intensity (50 µmol m^−2^ s^−1^, Rc50), the thermal-reversion related hypocotyl response becomes negligible, allowing us to isolate thermal-reversion-independent temperature effects^26^. Accordingly, under Rc50, the temperature-dependent decrease in PB number observed from 12 to 21 °C under Rc10 was abolished, confirming that this cooler-range effect depends on phyB thermal reversion (Fig. 5b, c)^31,50^. In contrast, PB thermosensitivity persisted from 21 to 27 °C even under Rc50, indicating a thermal-reversion-independent component that is consistent with regulation of seeding-site efficacy (Fig. 5b, c).

Together, these results show that PB thermosensitivity emerges from two separable inputs—nucleator state (phyB thermal reversion) and temperature-responsive seeding-site efficacy—whose relative contributions differ across PB site classes.

### Cell identity programs seeding-site efficacy

**Fig. 6:**
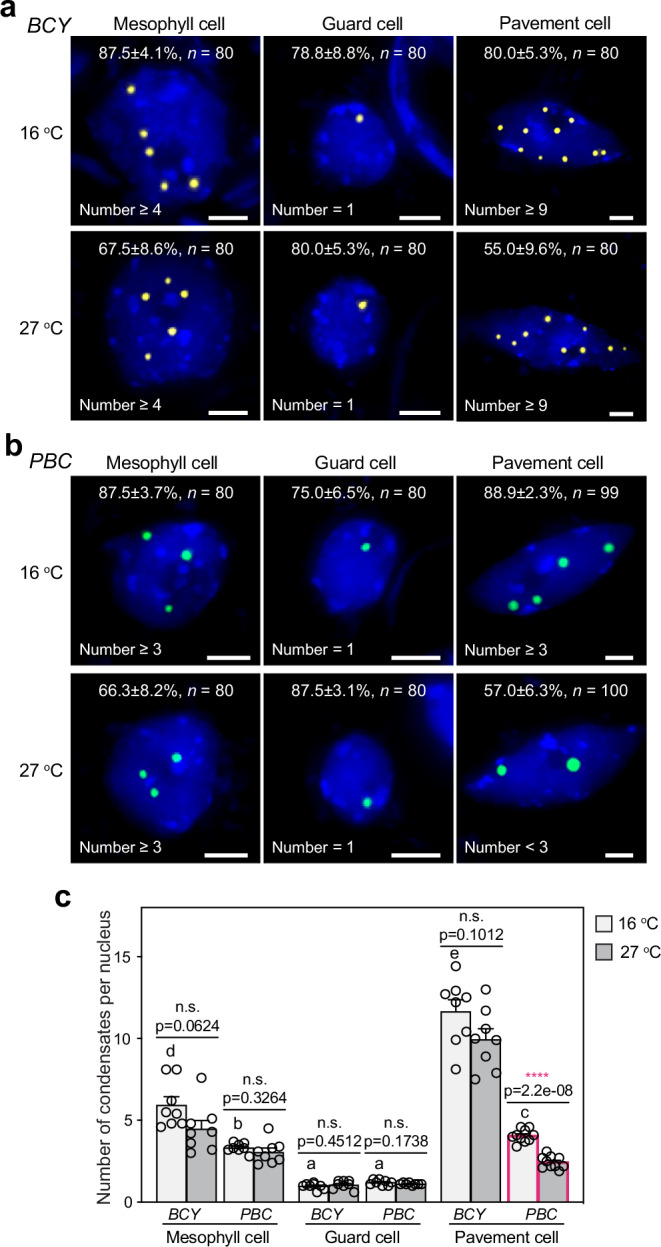
Seeding-site efficacy is cell-type specific. **a** Maximum-projection, deconvolved fluorescence microscopy images showing representative steady-state patterns of BCY bodies (OPM-YFP) in cotyledon mesophyll, guard, and pavement cell nuclei from 4-d-old *BCY* seedlings grown under continuous R light (10 µmol m^−2^ s^−1^) at 16 or 27 °C. Nuclei are counterstained with DAPI (blue). For each condition, the percentage of nuclei displaying the representative pattern is reported as mean ± s.e.m. across biological replicates; *n* denotes total nuclei scored. Scale bars, 2 μm. **b** As in (**a**), for *PBC* seedlings (phyB-CFP PBs) in the same three cell types at 16 or 27 °C under R light (10 µmol m^−2^ s^−1^). Nuclei are counterstained with DAPI (blue). The percentage of nuclei displaying the representative pattern is reported as mean ± s.e.m. across biological replicates; *n* denotes total nuclei scored. Scale bars, 2 μm. **c** Global occupancy (mean number of per nucleus) for BCY bodies and PBC PBs in the three cell types at 16 or 27 °C (conditions as in **a**, **b**). Bars show mean values and error bars indicate ± s.e.m. Within each genotype and cell type, temperature effects (16 vs 27 °C) are indicated by asterisks (two-sided Student’s *t*-test; **** *p* < 0.0001; n.s., not significant); exact *p* values and test statistics are provided in Source Data. BCY occupancy reports seeding-site efficacy, whereas PBC integrates nucleator state with seeding-site efficacy. For (**a**–**c**), biologi**c**al replicates were as follows: *BCY*, all three cell types (*n* = 8); *PBC*, mesophyll and guard cells (*n* = 8), and pavement cells (*n* = 10). Source data underlying quantification of BCY bodies and PBC PBs in mesophyll, guard, and pavement cell nuclei at 16 and 27 °C in (**a–c**) are provided in the Source Data file.

We next asked whether cell identity regulates PB seeding-site efficacy. We previously showed that the average number of PBs per nucleus varies across organs and cell types, implying cell-type-specific control of PB nucleation in *PBC*^31^. To isolate seeding-site efficacy from phyB conformational control, we quantified the global occupancy (mean number per nucleus) of BCY bodies in three cotyledon cell types: pavement, mesophyll, and guard cells. Mesophyll and guard cells exhibited markedly lower occupancy than pavement cells at both 16 and 27 °C (Fig. 6a, c). At 16 °C, BCY bodies decreased from ~11 per nucleus in pavement cells to ~6 in mesophyll cells and ~1 in guard cells (Fig. 6a, c). Moreover, the BCY occupancy was temperature-insensitive in all three cell types (16 vs 27 °C; Fig. 6c), indicating that baseline seeding-site efficacy is strongly cell-type programmed and, at the global level, largely stable across the 16-to-27 °C temperature range.

We then quantified the PBs in PBC under the same conditions. Mesophyll and guard cells also contained fewer PBs than pavement cells, but the reduction was less pronounced than for BCY (Fig. 6b, c), consistent with full-length phyB integrating nucleator state with site efficacy. Strikingly, unlike pavement cells, mesophyll and guard cells showed no warm-temperature reduction in global PB occupancy in *PBC* (16 vs 27 °C; Fig. 6c). This pattern suggests that thermosensitive seeding sites active in pavement cells are diminished or inactive in mesophyll and guard cells, leaving primarily thermostable sites operative. Together, these results show that both seeding-site efficacy and thermosensitivity are cell-type specific, enabling developmental programs to tune condensate number through regulation of site competence.

## Discussion

**Fig. 7:**
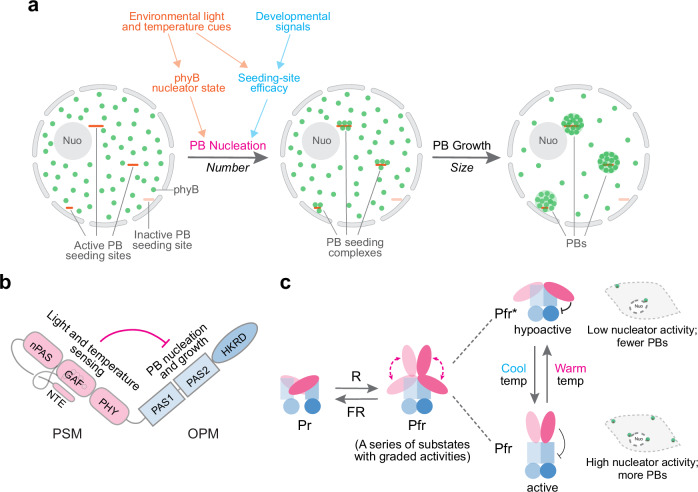
Model: condensate number is set by nucleator state and seeding-site efficacy. **a** Nucleation and growth are separable. PB formation initiates when phyB nucleates at defined seeding sites, which recruit phyB and raise its local concentration. PB number (per nucleus) is set by the overall probability of nucleation across sites, whereas PB size is determined by post-nucleation growth. At each site, nucleation depends on two inputs: (i) nucleator state (phyB conformation), which sets the output module (OPM) nucleation potency and (ii) seeding-site efficacy (site competence), which can be tuned by environmental cues and developmental signals, and can confer cell-type-specific activation or repression of individual sites. **b** Regulatory gating by the photosensory module (PSM). Domain schematic of phyB showing the PSM and OPM. PSM attenuates OPM activities; light- and temperature-dependent PSM conformations modulate OPM nucleation potency (controlling PB number) and OPM-driven growth (controlling PB size), enabling graded and reversible regulation. **c** Graded nucleator states. We propose that the active photostate Pfr comprises a series of substates in which PSM imposes graded inhibition on OPM. YHB marks an intermediate, hypoactive Pfr state (Pfr*), where nucleation is more strongly attenuated than in high-potency Pfr while growth remains permissive. Cool temperatures favor high-potency Pfr; warm temperatures enrich Pfr*. Thus, condensate number emerges from integrating nucleator state with site-specific seeding efficacy.

Membraneless organelles form through separable nucleation and growth steps. Our previous work showed that PBs nucleate nonrandomly at defined subnuclear locations and that temperature selectively modulates PB number without altering PB size^31,50^, making PBs a tractable in vivo system to interrogate nucleation control independently of growth (Fig. 7a). Here we show that phyB’s OPM is sufficient to nucleate the full repertoire of site-defined PB types and that the PSM functionally attenuates this nucleation activity, enabling environmental control through phyB conformational state (Fig. 7b, c).

The in vivo PB nucleation mechanism likely involves interactions between phyB and seeding sites that extend beyond the intramolecular interactions that drive OPM condensation^23,32,51,52^. Although OPM dimerization/oligomerization is necessary and sufficient for condensation in vivo and in vitro^23,32,51,52^, it was unclear whether OPM-only condensates correspond to the same site-defined PB types formed by full-length phyB. By mapping BCY bodies relative to chromocenters and the nucleolus, we show that OPM alone nucleates all twelve site-defined PB types, demonstrating that OPM can engage the full repertoire of competent seeding sites (Fig. 1).

Two decades of work established that OPM drives phyB nuclear accumulation and PB formation and that PSM-OPM contacts in Pr can mask OPM activities, with photoactivation to Pfr relieving these contacts and permitting nuclear accumulation, PB formation, and partner interactions^10,12,22,23,51^. Cryo-EM structures support this architecture by revealing extensive Pr-to-Pfr rearrangements that disrupt specific PSM-OPM interactions and enable alternative dimer interfaces^19–21^. Our results extend this model to the regulation of PB nucleation and reveal a nuance: even under conditions favoring Pfr, full-length phyB exhibits lower PB occupancy than OPM alone across all mapped seeding-site classes (Fig. 2), indicating residual PSM-mediated attenuation within the active-state ensemble. Consistently, increasing R light to saturating levels enhances PB occupancy, whereas warming under nonsaturating R light reduces it, showing that PB number can be quantitatively tuned by environmentally regulated phyB conformation (Fig. 5b, c).

Point mutations in OPM further dissect the OPM nucleation activity from PSM-mediated regulation. While A719V, A750V, G767R, E812K, and D1040V directly reduce OPM-driven nucleation, G674D has only a weak effect on OPM-only condensation despite its essential role in PB formation in the context of full-length phyB (Fig. 4b, c)^28,52^. These results provide genetic evidence that OPM’s intrinsic nucleation/condensation potency and PSM-mediated regulation can be separated.

Our data support a model in which active phyB comprises multiple substates with graded nucleation potency (Fig. 7c). Under nonsaturating R light, warming from 12 to 27 °C progressively reduces PB occupancy, consistent with temperature-dependent destabilization of the active state via thermal reversion. A simple two-state model would attribute this effect solely to decreased Pfr abundance at warm temperatures. However, the behavior of the constitutively active YHB allele argues that active phyB can differ qualitatively in nucleation potency. Although YHB forms PB constitutively, it shows markedly reduced PB occupancy across seeding-site classes compared to wild-type phyB (Fig. 3), indicating a hypoactive active state Pfr* that supports nuclear accumulation and PB growth but is deficient in nucleation (Fig. 7c). This provides a functional separation between nucleation and growth and motivates a graded-state model in which warming enriches a YHB-like Pfr* state at the expense of a higher-potency Pfr state. Spectroscopic heterogeneity reported for Cph1 phytochrome from the cyanobacterium *Synechocystis* sp. PCC6803 is consistent with the plausibility of multiple long-lived photostate populations^62,63^.

This interpretation differs from cryo-EM analyses reporting similar YHB and Pfr structures^20,21^. One possibility is that the cryo-EM YHB-PIF6 complex stabilizes a Pfr-like conformation, whereas in vivo, without stabilizing partners, YHB may populate a head-to-head active state with partial persistence of intramolecular contacts that selectively attenuate OPM nucleation. Defining the structural basis of Pfr-Pfr* interconversion is an important future direction.

In addition to the nucleator state, our results identify seeding-site efficacy as a major determinant of PB number. Because BCY lacks light/temperature sensing, its occupancy provides an operational readout of site competence under different conditions. Leveraging this property, we show that warming alters the efficacy of specific seeding-site classes in opposite directions—CC1 and nC decrease; CC5 increases—explaining why global PB counts can mask site-level responses (Fig. 5a). Moreover, cell identity strongly programs site competence. Within the same organ, pavement, mesophyll, and guard cells show striking differences in global OPM-YFP occupancy, and mesophyll and guard cells lose warm-induced reductions in full-length phyB-CFP PBs seen in pavement cells (Fig. 6c). These findings imply that developmental programs can modulate baseline seeding competence. Because CC-associated PBs localize near pericentromeric heterochromatin, a parsimonious hypothesis is that chromatin state and nuclear architecture tune seeding-site efficacy and its temperature responsiveness.

Our analyses primarily use fixed-sample imaging to quantify site-defined PB occupancy under sustained light and temperature regimes. This choice reflects both biological and technical considerations. Biologically, phyB functions as a principal environmental sensor that integrates irradiance and temperature over extended periods, and steady-state PB patterns correlate with phyB signaling outputs under continuous light conditions^12,28,31^. Technically, live-cell fluorescence imaging is challenging because excitation light can perturb phyB photostate and PB patterns^64,65^; maintaining stable temperature/light fields while collecting volumetric stacks across many nuclei limits throughput; moreover, BCY bodies (OPM alone) form constitutively regardless of light conditions. Fixed-sample imaging therefore provides a robust and scalable approach for quantifying PB positioning and abundance across large populations, enabling the site-class comparisons between OPM and full-length phyB as well as cell-type analyses central to our two-lever framework. A limitation is that steady-state occupancy is not equivalent to a kinetic nucleation rate; therefore, our conclusions focus on how nucleator state and seeding-site efficacy shape the steady-state distribution of PB number and site occupancy, and we do not infer absolute nucleation or dissolution rates. Direct measurements of nucleation kinetics would require establishing time-resolved 3D imaging after controlled light/temperature shifts with minimal perturbation of phyB photostate.

Together, our study defines intramolecular determinants of phyB PB nucleation and establishes a dual-lever framework in which PB number is set by integrating the nucleator state (phyB conformation) and site-specific efficacy, both environmentally and developmentally regulated. We anticipate that analogous integration of nucleator activity states with spatially patterned seeding competence may be a common strategy for spatiotemporal control of condensate number. Future investigations will focus on identifying the nuclear composition of PB seeding sites, investigating the role of PB clients in nucleation, understanding how chromatin context licenses nucleation, and resolving the structural basis of the Pfr-Pfr* transition.

## Methods

### Plant materials and growth conditions

The following Arabidopsis lines were previously reported: *BCY*^23^, *PBC*^23^, and *YHB*^53^. Transgenic lines expressing OPM-YFP (BCY) and OPM-YFP fused with an SV40 NLS (BCY-NLS) carrying individual OPM substitutions (*BCY*^*G674D*^, *BCY*^*A719V*^, *BCY*^*A750V*^, *BCY*^*G767R*^, *BCY*^*E812K*^) in the *phyB-9* background, and the corresponding NLS-tagged lines (*BCY*^*G674D*^*-NLS*, *BCY*^*A719V*^*-NLS*, *BCY*^*A750V*^*-NLS*, *BCY*^*G767R*^*-NLS*, *BCY*^*E812K*^*-NLS*) were generated in this study.

Seeds were surface-sterilized and plated on half-strength Murashige and Skoog (½ MS) medium with Gamborg’s vitamins (MSP06, Plant Cell Labs, Smithfield, UT) supplemented with 0.5 mM MES (pH 5.7) and 0.8% (w/v) Phytoagar (40100072-2, PlantMedia, Dublin, OH). Seeds were stratified for 5 days at 4 °C in darkness and then transferred to an LED growth chamber (Percival Scientific, Perry, IA). Seedlings were grown under continuous R light at either 10 μmol m^−2^ s^−1^ (Rc10) or 50 μmol m^−2^ s^−1^ (Rc50). For temperature treatments, stratified seeds were incubated for 48 h at 21 °C to induce germination and then shifted to 12, 16, or 27 °C for an additional 48 h. Light fluence rates were measured using an Apogee PS-200 spectroradiometer (Apogee Instruments, Logan, UT).

### Plasmid construction and generation of transgenic lines

PCR primers used for cloning are listed in Supplementary Table 1. Constructs encoding OPM-YFP (BCY) or OPM-YFP-NLS (BCY-NLS) with point mutations were generated by subcloning the C-terminal region of *PHYB* cDNA (amino acids 594-1172) carrying the indicated substitutions (G674D, A719V, A750V, G767R, E812K) into the KpnI site of pCHF3-YFP and pCHF3-YFP-NLS using HiFi DNA Assembly (New England Biolabs, Ipswich, MA). Mutation-containing primers were used with primers flanking the phyB C-terminal region; the template was the previously described BCY construct^52^. All transgenes were driven by the CaMV 35S promoter.

Transgenic lines were generated by transforming *phyB-9* with *Agrobacterium tumefaciens* strain GV3101 carrying the indicated pCHF3 constructs. For each construct, more than 10 independent T1 lines were selected on ½ MS medium containing 50 µg/ml kanamycin. T2 lines with single-locus insertions were identified by a 3:1 segregation ratio for kanamycin resistance, and homozygous T3 plants were used for the experiments.

For OPM dimerization assays, coding sequences corresponding to BCY amino acids 594-1172 (wild type or carrying G674D, A719V, A750V, G767R, E812K, or D1040V) were subcloned into pCMX-PL2-CterHA between KpnI and EcoRI using HiFi DNA Assembly.

### Design and synthesis of Oligopaints probe libraries

Oligopaints libraries were designed as described previously^50,66,67^. Each library comprises probes uniquely complementary to a defined genomic region. Probes contain a 36–41 nt genomic sequence flanked by non-genomic sequences: a universal reverse-transcription (RT) primer and library-specific forward/reverse index primers (see Supplementary Dataset 1 for probe sequences). *CEN178* and pericentromeric libraries (*PC1N*–*5N* and *PC1S*–*5S*) were designed with OligoMiner using default parameters^50,68^: **-l 36 -L 41 -g 20 -G 80 -t 42 -T 47 -X “AAAAA;TTTTT;CCCCC;GGGGG”**. The *CEN178* probes were designed using the pAL1 centromere sequence^69^. Probe sequences are provided in the Supplementary Dataset 1.

### Synthesis of Oligopaints probes

Oligopaints probes were generated as described previously^50^. In brief, Oligopaints probes were amplified from the oligopools (GenScript USA Inc., Piscataway, NJ) using forward and reverse index primers (primer sequences are listed in Supplementary Dataset 1). Double-stranded PCR products were used as templates for in vitro transcription with the HiScribe^®^ T7 Quick High Yield RNA Synthesis Kit (NEB). The resulting RNA was reverse transcribed by Maxima H Minus Reverse Transcriptase (EP0753, Thermo Fisher Scientific) with either fluorescently labeled RT primers (for directly labeled *CEN178* probes) or locked nucleic acid (LNA) RT primers (for branched-DNA amplification of pericentromeric probes). *CEN178* probes were labeled with ATTO 633 using a fluorescently labeled RT primer (Supplementary Dataset 1). Pericentromeric probe sets were synthesized using LNA RT primers (Supplementary Dataset 1). RNA templates were removed by RNase A treatment (37 °C for 1 h, 50 °C for 30 min, 92 °C for 15 min). RT primer incorporation was verified by 10% PAGE run in a 60 °C water bath for 30 min. Single-stranded DNA probes were purified using the DNA Clean & Concentrator-100 kit (Zymo Research, Irvine, CA).

### ImmunoFISH

Nuclear fixation and slide preparation were performed as described previously^50^. Seedlings were vacuum-infiltrated for 30 min in 4% (v/v) paraformaldehyde (15710, Electron Microscopy Sciences) with 5% DMSO in PBS. Fixation was quenched with three rinses in 50 mM NH₄Cl in PBS, followed by two washes in PBS. Approximately 100 cotyledons were chopped in 50 µL lysis buffer [15 mM Tris-HCl (pH 7.5), 50 mM EDTA, 0.5 mM spermine·4HCl, 80 mM KCl, 20 mM NaCl, 0.1% Triton X-100]. Homogenate was diluted 1:4 into nuclei suspension buffer [100 mM Tris-HCl (pH 7.5), 50 mM KCl, 2 mM MgCl₂, 5% sucrose, 0.05% Tween-20]. Nuclei suspensions were dispensed in 30 µL aliquots onto Superfrost^®^ Plus slides (Avantor, Radnor, PA) and air-dried for 4 h at room temperature. Slides were used immediately or stored at −20 °C.

ImmunoFISH was performed as previously described^50^. A 55 µL SecureSeal hybridization chamber (621505, Grace Bio-Labs, Bend, OR) was heat-activated and mounted onto slides containing fixed nuclei. Slides were washed in MgPBS (PBS + 1 mM MgCl₂) and permeabilized for 15 min in MgPBS containing 0.5% Triton X-100. After two 5-min rinses in MgPBS, slides were blocked for 30 min at room temperature in blocking solution (MgPBS containing 2% goat serum, 1.5% BSA, and 0.02% Tween-20). The nuclei were incubated with primary antibodies for 4 h at 37 °C in blocking solution: rabbit anti-GFP (A-11122, Thermo Fisher Scientific) or chicken anti-GFP (A10262, Thermo Fisher Scientific), 1:100, to detect OPM-YFP, phyB-CFP, and YHB-YFP; and mouse anti-fibrillarin (MA3-16771, Thermo Fisher Scientific), 1:100, to label nucleoli. Slides were washed three times in MgPBS containing 0.02% Tween-20 and incubated for 12 h at 37 °C with secondary antibodies. To detect phyB-CFP/YFP signals, the following secondary antibodies were used at 1:2000 dilution: goat anti-rabbit Alexa Fluor Plus 488 (A32731, Thermo Fisher Scientific) or Alexa Fluor 405 (A-31556, Thermo Fisher Scientific), and goat anti-chicken Alexa Fluor 488 (A-11039, Thermo Fisher Scientific) or Alexa Fluor Plus 405 (A-48260, Thermo Fisher Scientific). To detect fibrillarin, donkey anti-mouse Alexa Fluor 488 (A-21202, Thermo Fisher Scientific, Waltham, MA) was used at 1:2000 dilution. Slides were then washed twice and post-fixed for 15 min in 2% (v/v) paraformaldehyde in MgPBS, followed by two 5-min MgPBS washes.

For FISH pretreatment, nuclei were treated with 0.1 N HCl for 20 min, rinsed 5 min in sterile water, washed twice for 5 min in 2× SSCT (0.3 M NaCl, 30 mM sodium citrate, 0.1% Tween-20), and incubated for 3 min in 2× SSCT containing 50% formamide and 400 µg mL^−1^ denatured salmon sperm DNA. Slides were incubated at 60 °C for 20 min and cooled to room temperature. Genomic DNA was denatured at 92 °C for 2.5 min. Hybridization was performed overnight at 42 °C in hybridization buffer (2× SSCT, 50% formamide, 10% dextran sulfate, 40 ng/μL RNase A) containing Oligopaint probes: 0.2–0.3 pmol μL^−1^ fluorescent *CEN178* probes or 0.7–0.8 pmol μL^−1^ LNA pericentromeric probes. Slides were washed at room temperature 3x for 10 min in 2× SSCT. For branched-DNA amplification, slides were incubated sequentially in 50 μL 2× SSCT containing 0.5 pmol preamplifier (55 °C, 25 min), washed twice for 5 min, incubated with 0.5 pmol amplifier (55 °C, 25 min), washed twice for 5 min, incubated in 0.5 pmol fluorescent oligos (55 °C, 25 min), and washed twice for 10 min. Slides were mounted ProLong™ Gold Antifade (P10144, Thermo Fisher Scientific).

### Confocal microscopy and image analysis

Three-dimensional z-stacks of cotyledon pavement-cell nuclei were acquired on a Zeiss LSM 800 confocal microscope using a 100×/1.4 NA oil-immersion objective. Excitation/emission windows were: Alexa Fluor 405 (Ex 405 nm, Em 400–570 nm), ATTO 488/Alexa Fluor 488 (Ex 488 nm, Em 400–560 nm), ATTO 555 (Ex 561 nm, Em 560–630 nm), and ATTO 647 (Ex 640 nm, Em 645–700 nm). Z-stacks were acquired with 0.7 µm spacing. Maximum projections were generated in Zeiss ZEN 2.3 for analysis. Graphs were generated and statistical analyses were performed using GraphPad Prism 9 (GraphPad Software, San Diego, CA, USA). Two-sided Student’s *t*-tests were performed using Microsoft Excel v16.89.

PB types were classified by spatial relationship to nucleolar and *CEN178* signals. PBs visually overlapping with or directly contacting a *CEN178* signal were classified as chromocenter-associated (CC-PBs). Pericentromeric probes were used to distinguish five chromosome-specific CC classes (CC1-CC5). PBs spatially separated from *CEN178* signals were classified as non-chromocenter-associated (nC-PBs), yielding six CC-based classes. PBs were further subdivided by nucleolar association: PBs at the nucleolar periphery were classified as nucleolar-associated (Nuo), whereas PBs separated from the nucleolus were classified as non-nucleolar (nNuo), yielding 12 site-defined PB types.

For 3D visualization of CC2-associated PBs in BCY and PBC, raw z-stacks were deconvolved using Huygens Essential v19.10 (Scientific Volume Imaging, Hilversum, Netherlands) and rendered as surfaces using the Surface Render module. *CEN178* signals (red) and chromosome 2 pericentromeric signal (cyan) were rendered as solid volumes; phyB-CFP or OPM-YFP (yellow) labeled PBs. Representative images were exported as TIFF files and processed in Adobe Photoshop v26.11.2 (Adobe Inc., Mountain View, CA).

### Quantification of global PB number by fluorescence microscopy

Seedlings were fixed under vacuum for 10 min in 1% (v/v) paraformaldehyde (15710, Electron Microscopy Sciences) with 5% DMSO in PBS. Samples were quenched three times in 50 mM NH₄Cl in PBS, washed twice in PBS, permeabilized in 0.5% Triton X-100 in PBS for 20 min, and counterstained with DAPI (5 µg mL^−1^) for 25 min. After three PBS washes, seedlings were mounted in water with the cotyledon adaxial surface facing upward.

PB quantification was performed as described previously^64,65^. In brief, 3D stacks were acquired on a Zeiss Axio Observer Z1 inverted microscope equipped with a Plan-Apochromat 100x/1.4 NA oil-immersion objective and an Axiocam mono camera. Fluorescence was excited using an X-Cite 120LED Boost (Excelitas Technologies, Waltham, MA) and detected using Zeiss filter sets: DAPI (Filter Set 49; Ex 365 nm, Em 445/50); CFP (Filter Set 47 HE; Ex 436/25, Em 480/40); and YFP (Filter Set 46; Ex 500/25, Em 535/40). Z-stacks were collected at 0.25 µm intervals. Maximum projections were generated in Zeiss ZEN 2.3 and condensates were manually counted.

### In vitro protein translation and blue-native PAGE

Wild-type and mutated HA-tagged OPM proteins were synthesized in vitro using the TNT T7 Coupled Reticulocyte Lysate System (Promega, Madison, WI) according to the manufacturer’s instructions. Translation reactions were mixed with BN-PAGE sample buffer (1× NativePAGE™ sample buffer plus 0.25% NativePAGE™ Coomassie Blue G-250 additive) using the NativePAGE™ Sample Prep Kit (Thermo Fisher Scientific) and resolved on 4–16% NativePAGE™ Bis-Tris protein gels (Thermo Fisher Scientific). Electrophoresis was performed at 4 °C using Dark Blue Cathode Buffer at 30–40 V for 3 h, until the dye front migrated approximately one-third of the gel, followed by separation in Light Blue Cathode Buffer at 20–25 V overnight. Proteins were transferred to PVDF membrane at 70 V for 7 h at 4 °C. Membranes were briefly wetted in methanol (3 min), blocked with 1% milk in TBS, and incubated with primary antibody at 4 °C overnight, followed by HRP-conjugated secondary antibody. HA-tagged proteins were detected using rabbit anti-HA clone RM305 (MA5-27915, Thermo Fisher Scientific) at 1:1000, followed by goat anti-rabbit HRP (1706515, Bio-Rad) at 1:5000. Chemiluminescent signals were developed using SuperSignal™ West Dura Extended Duration Substrate (Thermo Fisher Scientific). Images of immunoblots were taken using a LI-COR Odyssey XF and quantified in LI-COR Image Studio (LI-COR, Lincoln, NE). Graphs were generated and statistical analyses were performed using GraphPad Prism 9 (GraphPad Software, San Diego, CA, USA). Uncropped and unprocessed scans of gels are provided in the Source Data file.

### Reporting summary

Further information on research design is available in the Nature Portfolio Reporting Summary linked to this article.

## Supplementary information

Supplementary Information

Description of Additional Supplementary Files

Supplementary Data 1

Reporting Summary

Transparent Peer Review file

## Source data

Source Data

## Data Availability

The *Arabidopsis* lines and information on the reagents used in the current study are available from the corresponding author upon request. Source data are provided with this paper.
