# Supplementary material for: Phytochrome B sets condensate number through graded nucleator states and seeding-site efficacy
Source: Nat Commun. 2026 Jun 2;17:7072. doi: 10.1038/s41467-026-73929-w (PMC13392045; doi:10.1038/s41467-026-73929-w)
Supplement: Supplementary file 1 — Supplementary Information [file 41467_2026_73929_MOESM1_ESM.pdf]

Supplementary Table 1. List of primers for vector construction.

Description	Sequence 5'–3'
Primers for generating point mutations in OPM	
BCY(G674/D)_F	ATTCGCTGTGGATGCCGGAGATTGCATCAATGGATGGAACGCTAAG
BCY(G674/D)_R	TCCGGCATCCACAGCGAATATAGG
BCY(A719/V)_F	CAATAAGCTTCTTTCTCGTGTTTTGAGAGGGGACGAGGAAAAGAA
BCY(A719/V)_R	ACGAGAAAGAAGCTTATTGACAG
BCY(A750/V)_F	CAGTTTTTGTGGTTGTGAATGTTTGTTCCAGCAAGGACTACTTGAA
BCY(A750/V)_R	ATTCACAACCACAAAAACTGCTTT
BCY(G767/R)_F	TTGTCGGCGTTTGTTTTGTTAGACAAGACGTTACTAGTCAGAAAAT
BCY(G767/R)_R	AACAAAACAAACGCCGACAATGT
BCY(E812/K)_F	GAGAACACGTGCTGCCTGAAATGGAACATGGCGATGGAAAAG
BCY(E812/K)_R	CAGGCAGCACGTGTTCTCGTCAG
Primers for generating BCY and BCY-NLS point mutation lines	
BCY_F	ggggacgagctcggtaccATGAGCCGGAGTCAGCCATGG
BCY_R	agttggtgttggagtaggATATGGCATCATCAGCAT
Primers for BN-PAGE to test OPM dimerization	
OPM_F	ctgtaccagatatcaggatcggtaccATGAGCCGGAGTCAGCCATGG
OPM_R	aatctggaacatcgtatgggtaATATGGCATCATCAGCAT
Primers for Y2H assay	
pBridge-phyBN-F	aagcaagcctcctgaaagATGGTTTCCGGAGTCGGGG
phyBN-linker-R	aggggtgggagttggtgttggagtaggTGCACCTAACTCATCAATCCCC
linker-BD-F	ccaacaccaactcccacccctATGAAGCTACTGTCTTCTATCGAACA
pBridge-Myc-R	aattagcttggctgcaggCAGGTCCTCCTCTGAGATCAG
GAD-PASA-F	atggccatggaggccagtgaattcATGAACTCTAAAGTTGTGGATGGTG
GAD-HKRD-R	attcatctgcagctcgagcCTAATATGGCATCATCAGCATC
