# Supplementary material for: Phytochrome B sets condensate number through graded nucleator states and seeding-site efficacy
Source: Nat Commun. 2026 Jun 2;17:7072. doi: 10.1038/s41467-026-73929-w (PMC13392045; doi:10.1038/s41467-026-73929-w)
Supplement: Supplementary file 2 — Description of Additional Supplementary Files [file 41467_2026_73929_MOESM2_ESM.pdf]

Description of Additional Supplementary Files

File Name: Supplementary Data 1

Description: Oligopaints FISH probe libraries.

This dataset includes amplification index primers, fluorescently labeled reverse transcription primers for *CEN178* probe labeling, and oligo sequences used for branched-DNA amplification, and Oligopaints probe sequences.
