# Supplementary material for: Phytochrome B sets condensate number through graded nucleator states and seeding-site efficacy
Source: Nat Commun. 2026 Jun 2;17:7072. doi: 10.1038/s41467-026-73929-w (PMC13392045; doi:10.1038/s41467-026-73929-w)
Supplement: Supplementary file 4 — Reporting Summary [file 41467_2026_73929_MOESM4_ESM.pdf]

nature portfolio

Corresponding author(s): Meng Chen

Last updated by author(s): May 16, 2026

Reporting Summary

Nature Portfolio wishes to improve the reproducibility of the work that we publish. This form provides structure for consistency and transparency in reporting. For further information on Nature Portfolio policies, see our Editorial Policies and the Editorial Policy Checklist.

Statistics

For all statistical analyses, confirm that the following items are present in the figure legend, table legend, main text, or Methods section.

n/a	Confirmed	
☐	☒	The exact sample size (*n*) for each experimental group/condition, given as a discrete number and unit of measurement
☐	☒	A statement on whether measurements were taken from distinct samples or whether the same sample was measured repeatedly
☐	☒	The statistical test(s) used AND whether they are one- or two-sided<br>*Only common tests should be described solely by name; describe more complex techniques in the Methods section.*
☐	☒	A description of all covariates tested
☒	☐	A description of any assumptions or corrections, such as tests of normality and adjustment for multiple comparisons
☐	☒	A full description of the statistical parameters including central tendency (e.g. means) or other basic estimates (e.g. regression coefficient) AND variation (e.g. standard deviation) or associated estimates of uncertainty (e.g. confidence intervals)
☐	☒	For null hypothesis testing, the test statistic (e.g. *F*, *t*, *r*) with confidence intervals, effect sizes, degrees of freedom and *P* value noted<br>*Give P values as exact values whenever suitable.*
☒	☐	For Bayesian analysis, information on the choice of priors and Markov chain Monte Carlo settings
☒	☐	For hierarchical and complex designs, identification of the appropriate level for tests and full reporting of outcomes
☒	☐	Estimates of effect sizes (e.g. Cohen's *d*, Pearson's *r*), indicating how they were calculated

Our web collection on statistics for biologists contains articles on many of the points above.

Software and code

Policy information about availability of computer code

Data collection	N/A
Data analysis	OligoMiner, Zeiss ZEN 2.3, GraphPad Prism 9, Huygens Essential v19.10 , Adobe Photoshop v26.11.2, Microsoft Excel v16.89, LI-COR Image Studio

For manuscripts utilizing custom algorithms or software that are central to the research but not yet described in published literature, software must be made available to editors and reviewers. We strongly encourage code deposition in a community repository (e.g. GitHub). See the Nature Portfolio guidelines for submitting code & software for further information.

Data

Policy information about availability of data

All manuscripts must include a data availability statement. This statement should provide the following information, where applicable:

- Accession codes, unique identifiers, or web links for publicly available datasets
- A description of any restrictions on data availability
- For clinical datasets or third party data, please ensure that the statement adheres to our policy

The condensate quantification data for Figs. 1c-e, 2a-b, 3b-d, 4b-c, 5a-c, and 6a-c, and immunoblotting data for 4d-e are provided in the Source Data file. The Arabidopsis lines and information on the reagents used in the current study are available from the corresponding author upon request.

Research involving human participants, their data, or biological material

Policy information about studies with human participants or human data. See also policy information about sex, gender (identity/presentation), and sexual orientation and race, ethnicity and racism.

Reporting on sex and gender	*Use the terms sex (biological attribute) and gender (shaped by social and cultural circumstances) carefully in order to avoid confusing both terms. Indicate if findings apply to only one sex or gender; describe whether sex and gender were considered in study design; whether sex and/or gender was determined based on self-reporting or assigned and methods used. Provide in the source data disaggregated sex and gender data, where this information has been collected, and if consent has been obtained for sharing of individual-level data; provide overall numbers in this Reporting Summary. Please state if this information has not been collected. Report sex- and gender-based analyses where performed, justify reasons for lack of sex- and gender-based analysis.*
Reporting on race, ethnicity, or other socially relevant groupings	*Please specify the socially constructed or socially relevant categorization variable(s) used in your manuscript and explain why they were used. Please note that such variables should not be used as proxies for other socially constructed/relevant variables (for example, race or ethnicity should not be used as a proxy for socioeconomic status). Provide clear definitions of the relevant terms used, how they were provided (by the participants/respondents, the researchers, or third parties), and the method(s) used to classify people into the different categories (e.g. self-report, census or administrative data, social media data, etc.) Please provide details about how you controlled for confounding variables in your analyses.*
Population characteristics	*Describe the covariate-relevant population characteristics of the human research participants (e.g. age, genotypic information, past and current diagnosis and treatment categories). If you filled out the behavioural & social sciences study design questions and have nothing to add here, write "See above."*
Recruitment	*Describe how participants were recruited. Outline any potential self-selection bias or other biases that may be present and how these are likely to impact results.*
Ethics oversight	*Identify the organization(s) that approved the study protocol.*

Note that full information on the approval of the study protocol must also be provided in the manuscript.

Field-specific reporting

Please select the one below that is the best fit for your research. If you are not sure, read the appropriate sections before making your selection.

- [x] Life sciences
- [ ] Behavioural & social sciences
- [ ] Ecological, evolutionary & environmental sciences

For a reference copy of the document with all sections, see nature.com/documents/nr-reporting-summary-flat.pdf

Life sciences study design

All studies must disclose on these points even when the disclosure is negative.

Sample size	Sample sizes were chosen based on (i) established practice in quantitative photobody imaging studies, (ii) effect sizes observed in pilot experiments, and (iii) feasibility of scoring large numbers of nuclei across multiple independent biological replicates while maintaining identical growth, staining, and imaging conditions. For imaging-based analyses, we prioritized independent biological replicates (typically 3–10 per condition, depending on the assay) and scored dozens to hundreds of nuclei per replicate to obtain stable estimates of mean occupancy/number per nucleus and to capture between-seedling/batch variation. For biochemical assays (e.g., BN–PAGE dimerization), we used at least three independent in vitro translation reactions/blots per variant, which was sufficient to resolve consistent differences among constructs. Exact replicate numbers and total nuclei scored for each experiment are reported in the figure legends and Source Data. These sample sizes produced reproducible effects with clear separation among conditions and supported the statistical tests used (two-sided t-tests and ANOVA).
Data exclusions	No data were excluded from analysis.
Replication	All key findings were reproduced in independent biological replicates, with replicate numbers reported in the figure legends and Source Data. For imaging-based quantification, seedlings were grown in independent batches, nuclei were scored from large populations per condition, and the same acquisition and scoring criteria were applied across experiments; representative images shown reflect the dominant pattern observed across replicates. For biochemical assays (e.g., BN–PAGE dimerization), results were confirmed in independent in vitro translation reactions and repeat blots. Where new transgenic lines were generated, multiple independent transformants were screened and representative lines with consistent phenotypes were selected for analysis. We are not aware of any results that failed replication or could not be reproduced.
Randomization	Seedlings were randomly picked for nuclei isolation. Nuclei were randomly picked for imaging analysis in FISH and immunoFISH experiments.
Blinding	N/A

Reporting for specific materials, systems and methods

We require information from authors about some types of materials, experimental systems and methods used in many studies. Here, indicate whether each material, system or method listed is relevant to your study. If you are not sure if a list item applies to your research, read the appropriate section before selecting a response.

Materials & experimental systems

n/a	Involved in the study
☐	☒ Antibodies
☒	☐ Eukaryotic cell lines
☒	☐ Palaeontology and archaeology
☒	☐ Animals and other organisms
☒	☐ Clinical data
☒	☐ Dual use research of concern
☐	☒ Plants

Methods

n/a	Involved in the study
☒	☐ ChIP-seq
☒	☐ Flow cytometry
☒	☐ MRI-based neuroimaging

Antibodies

Antibodies used	Rabbit anti-GFP (A-11122, Thermo Fisher Scientific), chicken anti-GFP (A10262, Thermo Fisher Scientific), mouse anti-fibrillarin (MA3-16771, Thermo Fisher Scientific), goat anti-rabbit Alexa Fluor Plus 488 (A32731, Thermo Fisher Scientific, goat anti-rabbit Alexa Fluor 405 (A-31556, Thermo Fisher Scientific), goat anti-chicken Alexa Fluor 488 (A-11039, Thermo Fisher Scientific), goat anti-chicken Alexa Fluor Plus 405(A-48260, Thermo Fisher Scientific), donkey anti-mouse Alexa Fluor 488 (A-21202, Thermo Fisher Scientific), rabbit anti-HA clone RM305 (MA5-27915, Thermo Fisher Scientific), and goat anti-rabbit HRP antibody (1706515, Bio-Rad).
Validation	The antibodies can be validated following the manufacturers' instructions.

Plants

Seed stocks	The PBC, YHB, BCY lines were previously described. The transgenic lines expressing OPM-YFP (BCY) carrying the individual mutations in the phyB-9 background, including BCYG674D,BCYA719V, BCYA750V, BCYG767R, BCYE812K and their corresponding NLS- tagged versions BCYG674D-NLS, BCYA719V-NLS, BCYA750V-NLS, BCYG767R-NLS, and BCYE812K-NLS were generated in this study.
Novel plant genotypes	N/A
Authentication	PBC, YHB, BCY lines can be genotyped using methods described in the references listed in the Methods section. The BCY and BCY-NLS lines carrying the indicated mutations were authenticated by PCR genotyping using the corresponding primers listed in Supplementary Table 1.
