# Supplementary material for: Phytochrome B sets condensate number through graded nucleator states and seeding-site efficacy
Source: Nat Commun. 2026 Jun 2;17:7072. doi: 10.1038/s41467-026-73929-w (PMC13392045; doi:10.1038/s41467-026-73929-w)
Supplement: Supplementary file 5 — Transparent Peer Review file [file 41467_2026_73929_MOESM5_ESM.pdf]

nature portfolio

Peer Review File

Phytochrome B sets condensate number through graded nucleator states and seeding-site efficacy

Corresponding Author: Professor Meng Chen

This file contains all reviewer reports in order by version, followed by all author rebuttals in order by version.

Version 0:

Reviewer comments:

Reviewer #1

(Remarks to the Author)
Du et al. propose a framework for controlling phyB photobody number by decomposing phyB into OPM/PSM modules, treating OPM as the nucleator and PSM as a light- and temperature-dependent gate, and integrating this with nucleus-specific seeding-site efficacy. While this modular, nucleation-centered view is conceptually appealing, the study relies largely on steady-state photobody counts and currently lacks sufficient quantitative and kinetic evidence. As written, the work is therefore not yet compelling as a general physical principle, but concentration–response and time-resolved measurements would substantially strengthen the mechanistic case and could make it suitable for Nature Communications.

Major comments
1. Clarify and strengthen the definition/visualization of the 12 seeding sites (Fig. 1).
The "12 site-defined" positions are difficult for non-specialists to interpret, and the photobody signal in these key images appears weak. Because this site classification underpins all downstream analyses, the authors should improve sensitivity and presentation (e.g., higher-S/N imaging, 3D acquisition/segmentation, and clear 3D renderings) to show the 12 sites more convincingly and intuitively.

2. Demonstrate concentration dependence expected for heterogeneous nucleation and tone down overstatements/generalizations.
If OPM nucleates via heterogeneous nucleation at defined seeds, nucleation should depend on nuclear OPM concentration, with a potential transition from seed-biased nucleation to bulk/random nucleation at sufficiently high expression. This could be tested directly (e.g., in a phyB knockout background) by titrating OPM levels and quantifying dose–response behavior and site restriction. These data would provide essential quantitative support for the central model.
Related to the lack of quantitative data, several statements appear overstated given the largely steady-state dataset and limited kinetic validation, including "decouple nucleation from growth" and "establish general rules for the spatiotemporal control of condensate number" (lines 74–75). The authors should substantially soften this language (e.g., "suggest potentially generalizable principles"). Finally, the statement "This dual-lever framework generalizes beyond plants" (line 244) is too strong as written and should be softened (e.g., "may/could generalize beyond plants").

3. Revise terminology conflating steady-state counts with kinetic rates (e.g. Fig. 1d).
Referring to "number per nucleus" as a "nucleation rate" is misleading: a rate is a kinetic quantity (events per unit time). The authors should revise wording (e.g., "steady-state photobody number") and/or add time-resolved measurements to justify rate-based terminology.

Reviewer #2

(Remarks to the Author)
The article investigates the molecular mechanism underlying phytochrome B (phyB) photobody nucleation in plant cells, examining how environmental factors such as temperature and photo-sensory regulation influence phyB nucleation. To address these questions, the authors utilise phyB mutants, phyB deletion constructs, and subnuclear markers to assess the position and number of phyB photobodies.

The key findings elucidate the molecular basis of phyB photobody nucleation and demonstrate how spatial positioning and environmental conditions modulate this process. Importantly, these insights have broader implications for understanding nucleation events of other nuclear proteins that form biomolecular condensates.

This work is highly novel, representing the first comprehensive study to dissect the mechanism of nuclear condensate formation in plants with such depth and precision. The approaches used are technically challenging, requiring sophisticated genetic tools and advanced imaging strategies, and the authors should be commended for the rigor and clarity of their experimental design. Their detailed methodology not only ensures reproducibility but also sets a benchmark for future studies in this emerging field. By uncovering how environmental cues and molecular architecture govern photobody nucleation, this research significantly advances our understanding of nuclear condensate formation in plant cells and provides a conceptual framework that extends beyond plant biology to other eukaryotic systems.

The conclusions are well supported by the presented results, and both the experimental design and data analysis are robust and appropriately executed.

General points for consideration:
- Do phyB-interacting factors (PIFs) also follow the same mechanism of nucleation and environmental regulation as phyB?
- Does in vitro or ex vivo (mammalian cells) phyB nucleation and seeding follow the same pattern as in plant cells?
- Does the absence of PIFs affect phyB nucleation and seeding as well as its environmental regulation?
- Is phyB nucleation, seeding, growth and number environmentally regulated on other plant cell types (stem, hypocotyl, root).
- How are the nuclear markers (chromocenters, nucleolus) affected by 1) phyB (presence, absence, active, inactive) and 2) environmental conditions?

Specific points:
- line 95: please justify the arbitrary cut off (0.2)
- Line 89 and 121 please how the Nuo-CC1 to Nuo-CC5 categories differ and what are the criteria for each category
- line 150: Refer to Figure 4b
- line 316: correct degrees (superscript)
- line 320: "and washed briefly" please provide more detail
- Figure 2b. “3-8 biological replicates” please indicate the exact number of biological replicates
- Figure 2b – performing one way ANOVA would be informative for the comparisons
- Figure 6- PSM and OPM - define in the figure caption.
- Expand on the significance of the findings in the plant and outside the field.
- Define acronyms in every figure caption

Version 1:

Reviewer comments:

Reviewer #1

(Remarks to the Author)

Reviewer #2

(Remarks to the Author)
Many thanks to the authors for addressing my comments.

Open Access This Peer Review File is licensed under a Creative Commons Attribution 4.0 International License, which permits use, sharing, adaptation, distribution and reproduction in any medium or format, as long as you give appropriate credit to the original author(s) and the source, provide a link to the Creative Commons license, and indicate if changes were made.
In cases where reviewers are anonymous, credit should be given to 'Anonymous Referee' and the source.
The images or other third party material in this Peer Review File are included in the article's Creative Commons license, unless indicated otherwise in a credit line to the material. If material is not included in the article's Creative Commons license and your intended use is not permitted by statutory regulation or exceeds the permitted use, you will need to obtain permission directly from the copyright holder.
To view a copy of this license, visit https://creativecommons.org/licenses/by/4.0/

Response to Reviewers

We thank both reviewers for their thorough and constructive evaluations. We have revised the manuscript extensively to improve clarity, strengthen mechanistic support, and address concerns regarding visualization, terminology, and scope of conclusions.

Major changes include: (1) substantial rewriting of the Introduction and Discussion to better frame the general problem of condensate-number control and to appropriately scope generalization; (2) addition of new results (new Fig. 4) identifying OPM missense mutations that distinguish intrinsic nucleation/condensation competence from regulatory coupling in full-length phyB; (3) improved visualization and explanation of site-defined PB classes, including new 3D rendering examples (Fig. 2b); (4) systematic replacement of "nucleation rate" with steady-state metrics ("occurrence frequency", "occupancy", "mean number per nucleus") where appropriate. A tracked-changes manuscript ("Du_manuscripted_tracked_changes") is included.

<u>Reviewer #1</u>

Du et al. propose a framework for controlling phyB photobody number by decomposing phyB into OPM/PSM modules, treating OPM as the nucleator and PSM as a light- and temperature-dependent gate, and integrating this with nucleus-specific seeding-site efficacy. While this modular, nucleation-centered view is conceptually appealing, the study relies largely on steady-state photobody counts and currently lacks sufficient quantitative and kinetic evidence. As written, the work is therefore not yet compelling as a general physical principle, but concentration–response and time-resolved measurements would substantially strengthen the mechanistic case and could make it suitable for Nature Communications.

Major comments
1. Clarify and strengthen the definition/visualization of the 12 seeding sites (Fig. 1).
The "12 site-defined" positions are difficult for non-specialists to interpret, and the photobody signal in these key images appears weak. Because this site classification underpins all downstream analyses, the authors should improve sensitivity and presentation (e.g., higher-S/N imaging, 3D acquisition/segmentation, and clear 3D renderings) to show the 12 sites more convincingly and intuitively.

Response:

We agree that the 12 site-defined classes are central and should be more intuitive to non-specialists. We have strengthened both the rationale and the presentation.

Our scoring is based 3D z-stacks followed by classification using projected images, a standard approach in spatial genome/subnuclear organization analyses [Finn et al. (2017) *Methods*, doi: 10.1016/j.ymeth.2017.01.007] and consistent with our prior definition of the 12 PB classes [Du et al. (2024) *Nat Commun*, 10.1038/s41467-024-47789-1]. Association

was defined as visual overlap or direct contact between PB and landmark signals, scored across populations of 30-227 nuclei per PB class/condition.

We added side-by-side 3D surface renderings comparing a representative CC2-associated condensate in OPM-YFP (BCY) and a CC2-associated PB in full-length phyB (PBC) (new Fig. 1b), with insets to highlight the condensates.

2. Demonstrate concentration dependence expected for heterogeneous nucleation and tone down overstatements/generalizations.
If OPM nucleates via heterogeneous nucleation at defined seeds, nucleation should depend on nuclear OPM concentration, with a potential transition from seed-biased nucleation to bulk/random nucleation at sufficiently high expression. This could be tested directly (e.g., in a phyB knockout background) by titrating OPM levels and quantifying dose–response behavior and site restriction. These data would provide essential quantitative support for the central model.
Related to the lack of quantitative data, several statements appear overstated given the largely steady-state dataset and limited kinetic validation, including "decouple nucleation from growth" and "establish general rules for the spatiotemporal control of condensate number" (lines 74–75). The authors should substantially soften this language (e.g., "suggest potentially generalizable principles"). Finally, the statement "This dual-lever framework generalizes beyond plants" (line 244) is too strong as written and should be softened (e.g., "may/could generalize beyond plants").

Response:

We appreciate the thoughtful questions and agree that concentration-response and time-resolved measurements can be informative.

Empirical evidence against "random nucleation at high expression". In our prior work [Kim et al. (2024) *Nat Commun*, 10.1038/s41467-024-47790-8], we generated multiple lines phyB-CFP complementation lines spanning 7- to 65-fold endogenous phyB abundance. PB size increased with phyB-CFP abundance, but PB number in epidermal pavement cells remained similar across lines, arguing that increased expression does not produce a transition to "random" PB nucleation. Moreover, in our PB site-mapping study [Du et al. (2024) *Nat Commun*, 10.1038/s41467-024-47789-1], we used the *PBC* line with 65-fold phyB overexpression and still observed PB positioning at 12 distinct site classes. The following is from the abstract: "*We show surprisingly that even in PHYB overexpression lines – where PHYB condensation would be more likely to occur randomly – PBs positioned at twelve distinct subnuclear locations distinguishable by chromocenter and nucleolus landmarks, suggesting that PHYB condensation occurs nonrandomly at preferred seeding sites.*"

Our working hypothesis is that the effective nucleation threshold at preferred seeding sites in vivo is substantially lower than the threshold required for bulk, random nucleation. In

this view, once site-defined PBs form, they act as sinks that partition phyB into the condensation phase and buffer the nucleoplasmic (dilute-phase) concentration near the site-specific threshold. As a result, nucleoplasmic phyB may rarely, if ever, rise to the higher concentration needed for global random nucleation, which could explain why we do not observe a transition to random PB formation even at elevated phyB abundance. An instructive analogy is actin polymerization: actin monomers polymerize in vitro only above a relatively high concentration, whereas in cells, nucleation and localization factors lower the effective threshold and restrict filament assembly to specific sites, thereby repressing ectopic polymerization.

Steady-state focus and practical constraints. We clarified why steady-state PB patterns are informative and widely used in the phyB field: phyB functions as a sensor of sustained irradiance, and the steady-state PB patterns correlate with phyB output [Chen et al. (2003) *PNAS*, 10.1073/pnas.1935989100; Du et al. (2026) *Plant Physiol*, 10.1093/plphys/kiag058]. In addition, repeated imaging under excitation light can perturb phyB localization, making kinetic experiments under light/temperature transitions technically challenging. Therefore, fixed-sample imaging is commonly used for rigorous quantification [Du et al. (2024) *Methods in Mol Biol*, 10.1007/978-1-0716-3814-9_10]. We also cite prior work showing that warm temperature can remodel PB patterns on hours timescales [Hahm et al. (2020) *Nat Commun*, 10.1038/s41467-020-15526-z].

New genetic evidence supporting the separability of nucleation potency and regulation. We added new results (Fig. 4) showing that OPM mutations can preferentially impair intrinsic OPM condensation competence (A719V, A750V, G767R, E812K, and D1040V) versus primarily affecting full-length phyB PB formation despite weak effects on OPM-only condensation (G674D). Together with YHB (Fig. 3), these data strengthen the conclusion that OPM condensation potency and PSM-mediated regulation can be genetically separated.

Tone-done generalization. We revised language throughout to avoid overstatement.

3. Revise terminology conflating steady-state counts with kinetic rates (e.g., Fig. 1d). Referring to “number per nucleus” as a “nucleation rate” is misleading: a rate is a kinetic quantity (events per unit time). The authors should revise wording (e.g., “steady-state photobody number”) and/or add time-resolved measurements to justify rate-based terminology.

Response:

We agree. We have replaced “nucleation rate” with “occurrence frequency”, “steady-state occupancy”, or “mean number per nucleus” throughout the text, figures, and legends, to avoid implying kinetic measurements.

Reviewer #2:

The article investigates the molecular mechanism underlying phytochrome B (phyB) photobody nucleation in plant cells, examining how environmental factors such as temperature and photo-sensory regulation influence phyB nucleation. To address these questions, the authors utilise phyB mutants, phyB deletion constructs, and subnuclear markers to assess the position and number of phyB photobodies.

The key findings elucidate the molecular basis of phyB photobody nucleation and demonstrate how spatial positioning and environmental conditions modulate this process. Importantly, these insights have broader implications for understanding nucleation events of other nuclear proteins that form biomolecular condensates.

This work is highly novel, representing the first comprehensive study to dissect the mechanism of nuclear condensate formation in plants with such depth and precision. The approaches used are technically challenging, requiring sophisticated genetic tools and advanced imaging strategies, and the authors should be commended for the rigor and clarity of their experimental design. Their detailed methodology not only ensures reproducibility but also sets a benchmark for future studies in this emerging field. By uncovering how environmental cues and molecular architecture govern photobody nucleation, this research significantly advances our understanding of nuclear condensate formation in plant cells and provides a conceptual framework that extends beyond plant biology to other eukaryotic systems.

The conclusions are well supported by the presented results, and both the experimental design and data analysis are robust and appropriately executed.

We thank the reviewer for the positive assessment and the thoughtful questions.

General points for consideration:
- Do phyB-interacting factors (PIFs) also follow the same mechanism of nucleation and environmental regulation as phyB?

Response:

The current data indicate that PBs are scaffolded by phyB and PIFs behave as clients whose PB localization depends on active phyB in vivo [Du et al. (2026) *Plant Physiol*, 10.1093/plphys/kiag058]. While PIFs can exhibit condensation-like behavior in vitro or heterologous settings, PIF localization to PBs in vivo depends on the presence of active phyB in Arabidopsis. Our study focuses on phyB-driven PB nucleation in vivo.

- Does in vitro or ex vivo (mammalian cells) phyB nucleation and seeding follow the same pattern as in plant cells?

Response:

This is a very interesting question. It was shown that phyB can form cytoplasmic condensates in mammalian cells [Chen et al. (2002) Mol Cell, 10.1016/j.molcel.2022.05.026]. While phyB/OPM condensation in mammalian cells demonstrates intrinsic condensability, mammalian cells likely lack the seeding sites in Arabidopsis nuclei. In vivo nucleation properties should be assessed only in natural biological settings and are unlikely to be replicated in vitro or in heterogeneous systems.

- Does the absence of PIFs affect phyB nucleation and seeding as well as its environmental regulation?

Response:

This is an important question. Our current work establishes a quantitative framework for measuring site-defined PB occupancy; testing how specific clients (including PIFs) influence nucleation is now feasible and represents a clear direction for future work. We added this point explicitly to the Discussion.

- Is phyB nucleation, seeding, growth and number environmentally regulated on other plant cell types (stem, hypocotyl, root).

Response:

We previously showed that PB number varies between organs even within the same broad cell type (e.g., cotyledon vs hypocotyl epidermis) [Hahm et al. (2020) *Nat Commun*, 10.1038/s41467-020-15526-z]. In the present study, we focused on three cotyledon cell types (pavement, mesophyll, guard) to cleanly test the principle that cell identity programs seeding-site efficacy. Based on the concept, it would be interesting to expand the study to root cells (which have more complex cell types) in the future.

- How are the nuclear markers (chromocenters, nucleolus) affected by 1) phyB (presence, absence, active, inactive) and 2) environmental conditions?

Response:

Chromocenter compaction is influenced by light during seeding development [Bourbousse et al. (2015) *PNAS* 10.1073/pnas.1503512112], but those studies relied largely on DAPI-defined heterochromatin domains. Our site mapping used centromeric positioning (CEN178 FISH), which provides a more precise landmark. In our previous study, we showed that nucleolar association patterns track CC-nucleolus proximity [Fig. 3d, Du et al. (2024) *Nat Commun*, 10.1038/s41467-024-47789-1].

Specific points:
- line 95: please justify the arbitrary cut off (0.2)

Response:

In our previous study, we showed that the estimated random association between a PB and a CC is expected to colocalize in 15.7% of nuclei [Du et al. (2024) *Nat Commun*, 10.1038/s41467-024-47789-1]. We arbitrarily define 0.2 PB per nucleus, which means the presence of a site-defined PB in 20% nuclei, as the cut-off to separate frequent vs rare PB classes. It is important to note that key conclusions do not depend on this threshold.

- Line 89 and 121 please how the Nuo-CC1 to Nuo-CC5 categories differ and what are the criteria for each category

Response:

We clarified in text and legends that CC identity (CC1-CC5) is assigned using chromosome-specific pericentromeric Oligopaints, and "Nuo" indicates direct adjacency/overlap with the nucleolus.

- line 150: Refer to Figure 4b

Response:

Corrected

- line 316: correct degrees (superscript)

Response:

We have corrected the degree symbol.

- line 320: "and washed briefly" please provide more detail

Response:

We provided more details in the Methods.

- Figure 2b. "3-8 biological replicates" please indicate the exact number of biological replicates

Response:

Updated the figure legend to list exact biological replicate numbers for each condition.

- Figure 2b – performing one way ANOVA would be informative for the comparisons

Response:

Our primary comparison in Fig. 2b is between genotypes within each PB type, and we used two-tailed t-tests. We clarified this rationale in the Methods/legend. (If preferred, we can additionally report a two-way ANOVA genotype × type as a complementary analysis; however, it does not change conclusions.

- Figure 6- PSM and OPM - define in the figure caption.

Response:

Added definitions.

- Expand on the significance of the findings in the plant and outside the field.

Response:

We substantially rewrote the Discussion to emphasize broader condensate-number control and the relationship between phyB structure and nucleation.

- Define acronyms in every figure caption

Response:

We made the changes.
